# Exosomal mRNAs for Angiogenic–Osteogenic Coupled Bone Repair

**DOI:** 10.1002/advs.202302622

**Published:** 2023-10-17

**Authors:** Yifan Ma, Lili Sun, Jingjing Zhang, Chi‐ling Chiang, Junjie Pan, Xinyu Wang, Kwang Joo Kwak, Hong Li, Renliang Zhao, Xilal Y. Rima, Chi Zhang, Anan Zhang, Yutong Liu, Zirui He, Derek Hansford, Eduardo Reategui, Changsheng Liu, Andrew S. Lee, Yuan Yuan, Ly James Lee

**Affiliations:** ^1^ Department of Biomedical Engineering The Ohio State University Columbus OH 43210 USA; ^2^ William G. Lowrie Department of Chemical and Biomolecular Engineering The Ohio State University Columbus OH 43210 USA; ^3^ Key Laboratory for Ultrafine Materials of Ministry of Education and Frontiers Science Center for Materiobiology and Dynamic Chemistry East China University of Science and Technology 200237 Shanghai P. R. China; ^4^ Spot Biosystems Ltd. Palo Alto 94305 United States; ^5^ Department of Orthopedic Surgery and Shanghai Institute of Microsurgery on Extremities Shanghai Jiao Tong University Affiliated Sixth People's Hospital 200233 Shanghai China; ^6^ College of Pharmacy The Ohio State University Columbus OH 43210 USA; ^7^ School of Chemical Biology and Biotechnology Peking University Shenzhen Graduate School 518055 Shenzhen China; ^8^ Institute for Cancer Research Shenzhen Bay Laboratory 518055 Shenzhen China

**Keywords:** PEGylated poly (glycerol sebacate) acrylate (PEGS‐A) hydrogel, small extracellular vesicles carrying therapeutic mRNAs and associated microRNAs, track‐etched membrane‐based nanoelectroporation (TM‐nanoEP), vascular endothelial growth factor A (VEGF‐A), bone morphogenetic protein 2 (BMP‐2) mRNAs coupled angiogenic–osteogenic regeneration

## Abstract

Regenerative medicine in tissue engineering often relies on stem cells and specific growth factors at a supraphysiological dose. These approaches are costly and may cause severe side effects. Herein, therapeutic small extracellular vesicles (t‐sEVs) endogenously loaded with a cocktail of human vascular endothelial growth factor A (VEGF‐A) and human bone morphogenetic protein 2 (BMP‐2) mRNAs within a customized injectable PEGylated poly (glycerol sebacate) acrylate (PEGS‐A) hydrogel for bone regeneration in rats with challenging femur critical‐size defects are introduced. Abundant t‐sEVs are produced by a facile cellular nanoelectroporation system based on a commercially available track‐etched membrane (TM‐nanoEP) to deliver plasmid DNAs to human adipose‐derived mesenchymal stem cells (hAdMSCs). Upregulated microRNAs associated with the therapeutic mRNAs are enriched in t‐sEVs for enhanced angiogenic–osteogenic regeneration. Localized and controlled release of t‐sEVs within the PEGS‐A hydrogel leads to the retention of therapeutics in the defect site for highly efficient bone regeneration with minimal low accumulation in other organs.

## Graphical abstract

This study describes an easily scalable and cost‐effective method to produce abundant therapeutic small extracellular vesicles (t‐sEVs) carrying a cocktail of functional mRNAs (VEGF‐A and BMP‐2) within a customized injectable PEGylated poly (glycerol sebacate) acrylate (PEGS‐A) hydrogel cage for coupling bone regeneration in rats that have challenging femur critical‐size defects.

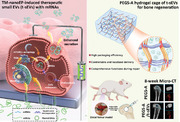

## 1 Introduction

Bony injuries (fractures), particularly critical‐size defects are unable to self‐heal and require orthopedic surgery and therapeutics.^[^ ^1^ ^]^ Bone tissue engineering (BTE) strategies such as growth factors and stem‐cell therapies have been widely explored in clinical trials. Several growth factors, such as recombinant human bone morphogenetic proteins (rhBMPs) and platelet‐derived growth factor (PDGF) are approved for use in the United States and Europe.^[^ ^2^ ^]^ However, the clinical and preclinical efficacy of these molecules has been disappointing, and adverse side effects have led to tight constraints imposed during clinical use or even withdrawal from the market (e.g., rhBMP‐2 and rhBMP‐7).^[^ ^3^ ^]^ The lack of desirable regenerative therapeutics in orthopedics renders current tissue engineering strategies challenging to meet patient demands.

The safety and translation efficiency of chemically modified mRNAs by in vitro synthesis have been demonstrated as vaccines in the coronavirus disease 2019 (COVID‐19) (SARS‐CoV‐2) pandemic. This success has prompted widespread interest in using mRNAs for various vaccination applications and other medical needs, including regenerative medicines.^[^ ^4^ ^]^ The mRNA‐related therapies are led by the construction optimization of synthetic mRNAs and using lipid or polymer nanoparticle (LNP or PNP) carriers for encapsulation and delivery.^[^ ^5^ ^]^ This approach, however, faces substantial challenges, including cytotoxicity and allergenicity (e.g., anaphylaxis and other manifestations of hypersensitivity) that have been found in some COVID‐19‐vaccinated populations.^[^ ^6^ ^]^ Therefore, more efficient and safer mRNA therapies are needed.

Extracellular vesicles (EVs), in particular small EVs (sEVs)—also referred to as “exosomes”—are typically 30–150 nm in diameter and originate from the endosomal pathway.^[^ ^7^ ^]^ These cell‐derived nanoparticles have emerged as promising regenerative agents because they carry a variety of native nucleic acids and proteins from parental cells.^[^ ^8^ ^]^ Their high biocompatibility, low immunogenicity, and intrinsic homing ability to cross physiological barriers make those cell‐secreted particulates a desirable alternative to LNP/PNP‐based synthetic mRNA systems.^[^ ^9^ ^]^ To enable specific mRNA packing and enhance exosome production, several attempts have been made to load full transcript mRNAs into cell‐secreted extracellular vesicles for treating central nervous system (CNS) diseases. For example, Ryosuke et al.^[^ ^10^ ^]^ developed a novel EXOsomal Transfer Into Cells (EXOtic) device by genetically engineering parental cells with specific encoding candidates, including six‐transmembrane epithelial antigen of the prostate 3 (STEAP3), syndecan‐4 (SDC4), and a fragment of L‐aspartate oxidase (NadB), to simultaneously boost the exosome yield and specific mRNA packing for Parkinson's disease treatment. In another case, Yang et al.^[^ ^11^ ^]^ introduced a cellular nanoporation (CNP) technology based on a sophisticated silicone‐based platform to achieve up to 50‐fold increase of the exosome yield and ≈1000‐fold enhancement in phosphatase and tensin homolog (PTEN) mRNA encapsulation for the treatment of brain cancer. While these approaches are sophisticated and effective for mRNA encapsulation in EVs, they often rely on conditional transfection and specialized microelectromechanical systems (MEMS) fabrication, which render them time‐consuming and less cost‐effective.

In comparison to those complicated and costly technologies, herein, we report a facile and accessible cellular nanoelectroporation system based on commercially available track‐etched membranes (TM‐nanoEP), that can produce abundant sEVs loaded with therapeutic mRNAs for bone tissue regeneration. By delivering a BMP‐2 and VEGF‐A plasmid cocktail to human adipose‐derived mesenchymal stem cells (hAdMSCs), the optimized coupling of human bone morphogenetic protein 2 (BMP‐2) and human vascular endothelial growth factor A (VEGF‐A) mRNAs in sEVs results in a synergistic angiogenic–osteogenic effect much stronger than using the synthetic mRNA/LNP formulation. We further demonstrate that the plasmid transfection enhances the expression of certain native microRNAs (miRNAs) associated with the transcribed mRNAs in sEVs. Delivery of these therapeutic sEVs (t‐sEVs) with enriched exosomal mRNAs and associated miRNAs in a customized injectable PEGylated poly (glycerol sebacate) acrylate (PEGS‐A) hydrogel enables localized and controlled release of therapeutics, leading to efficient bone regeneration in rats with challenging femur critical‐size defects.

## 2 Results

### 2.1 Track‐Etched Membrane‐Based Nanoelectroporation (TM‐nanoEP) for Mass Production of Therapeutic Extracellular Vesicles (EVs)

**Figure 1:**
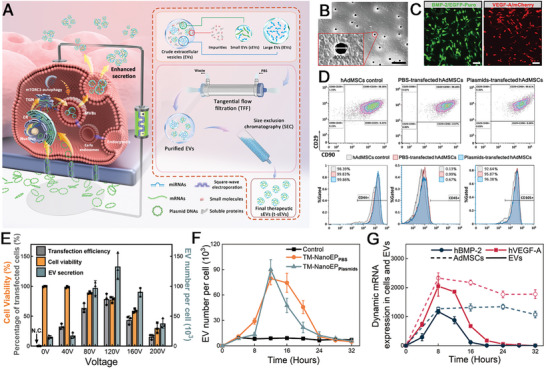
Track‐etched membrane‐based nanoelectroporation (TM‐nanoEP) for mass production of therapeutic extracellular vesicles (tEVs) loaded with enriched genetic materials. A) A schematic of TM‐nanoEP process for the production of EVs carrying therapeutic nucleic acids. These RNAs are transcribed from transfected plasmid DNAs (pDNAs) and reside within intraluminal vesicles (ILVs) inside multivesicular bodies (MVBs). The TM‐nanoEP induces non‐endocytic delivery of pDNAs and initiates mTORC1‐autophagic activities, which enhances the secretion of tEVs. Subsequent purification stages through tangential flow filtration (TFF) and size exclusion chromatography (SEC) isolate small EVs (sEVs) with optimized therapeutics from crude EVs. B) Scanning electron microscopy (SEM) image of track‐etched membrane consisting of nanopores with 400 nm in diameter (inset) (Scale bar: 5 µm). C) Fluorescent microscopic images of hAdMSCs adhered on the transparent track‐etched membrane with EGFP‐expressing human BMP‐2 (BMP2/EGFP‐Puro) and mCherry‐expressing human VEGF‐A (VEGF‐A/mCherry). The images were taken at 6 h after the TM‐nanoEP at 120 V (Scale bar: 100 µm). D) Flow cytometric profiles of MSCs‐defining markers CD29+/CD44+/CD45–/CD90+/CD105+ to identify the phenotype of hAdMSCs after TM‐nanoEP process. E) Transfection efficiency, cell viability, and EV production after the electroporation of hAdMSCs with the EGFP/Puro‐expressing plasmid under different voltages (*n* = 3 for all conditions). The EGFP/Puro‐expression in hAdMSCs and EV number increased with increasing voltage when the voltage was below 120 V and peaked at 120 V with a cell viability of 75%. The transfection efficiency, EV number, and cell viability decreased when the voltage was above 160 V. F) Dynamic profiles of EV release with medium collected and replaced every 4 h (*n* = 3 for each time point. N.C.: negative control of untreated cells). Significant EV generation was in the initial 24 h after TM‐nanoEP. G) Dynamic profiles of mRNA in parental hAdMSCs (dash curves) and derived EVs (solid curves) after TM‐nanoEP. All data are presented as mean ± SD.

Gene therapy using extracellular vesicles (EVs) as carriers is a promising approach for bone tissue engineering; however, insufficient EVs and inefficient encapsulation of therapeutic genes, particularly large‐size mRNAs, make it challenging to realize EV‐gene therapy in clinic uses.^[^ ^12^ ^]^ To address these limitations, we placed a monolayer of hAdMSCs on a commercial track‐etched membrane (embedded with nanopores 400 nm in diameter) and loaded a BMP‐2 and VEGF‐A plasmid cocktail in PBS buffer under the membrane (**Figure** **1**A,B). By applying an electric field across the membrane, the coupled BMP‐2/VEGF‐A plasmids encoded with reporter genes (EGFP‐Puro and mCherry, respectively) were non‐endocytically delivered into the cells via nano‐sized pores on the membrane. Strong expressions of green and red fluorescence were observed in the transfected cells after 6 h incubation (Figure 1C; Figure S1A, Supporting Information). The transfected cells were further evaluated by flow cytometry and morphology observation. They exhibited similar cell spreading and percentage of CD29^+^/CD44^+^/CD45^–^/CD90^+^/CD105^+^ after 24 h incubation when compared to the untreated hAdMSCs (Figure 1D; Figure S1B, Supporting Information). This suggests that the EVs originated from the parental cells without phenotypic changes in the TM‐nanoEP process. We then evaluated the cell viability, transfection efficiency, and EV secretion after TM‐nanoEP under different voltages (from 40 to 200 V) using the BMP‐2 plasmid with EGFP/Puro marker encoded as the delivery cargo. The transfected hAdMSCs were incubated in an exosome‐depleted medium for 24 h. A gentle voltage (40 V) led to >95% cell viability but a low transfection rate and poor EV production (Figure 1E). At higher voltages, both EV number and cell transfection efficiency increased with a peak at 120 V (>75% transfected hAdMSCs and approximately tenfold higher EV number per cell when compared to the untreated cells). Too high a voltage (e.g., 160 and 200 V) impaired cell responses and EV secretion (Figure 1E; Figure S1B, Supporting Information). Enhanced EV secretion mainly occurred between 8 and 20 h, and peaked at 12 h after TM‐nanoEP with or without plasmid delivery (Figure 1F). The transcribed mRNAs increased significantly in both transfected cells and secreted EVs at 4 h after TM‐nanoEP and reached a peak at 8 h. Notably, the transfected cells reached a plateau of mRNA expression after the peak time and maintained a high expression level for >32 h, while the transcript mRNAs encapsulated in EVs fell rapidly after the peak‐time (Figure 1G). Based on the EV secretion and mRNA encapsulation profiles, the collection period of therapeutic EVs was set from 0 to 16 h after TM‐nanoEP.

### 2.2 Characterization of EVs and Enriched RNAs in Therapeutic sEVs (t‐sEVs)

**Figure 2:**
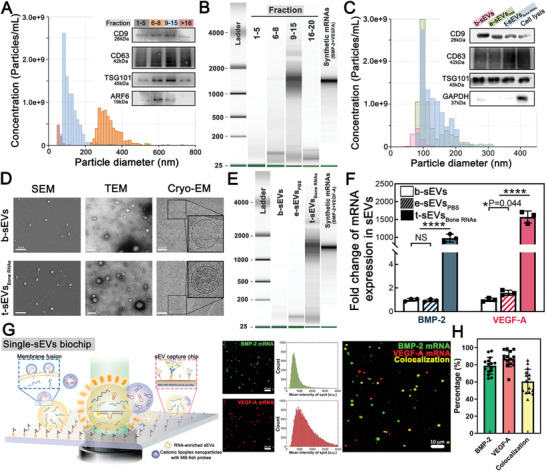
Characterization of EVs and enriched RNAs in small EVs (sEVs). A) Surface markers (CD9, CD63, TSG101, and ARF6) and particle size distributions of EV subpopulations after purification and isolation by tangential flow filtration (TFF) and size exclusion chromatography (SEC). B) RNA distribution in different fractions of EVs. C) Particle size distributions and surface markers of isolated blank sEVs (b‐sEVs), engineered sEVs (sEVs_PBS_)_,_ and therapeutic sEVs (t‐sEVs_Bone RNAs_) generated from blank hAdMSCs and hAdMSCs transfected by PBS buffer or bone‐related pDNA cocktail, respectively. D) SEM, transmission electron microscopy (TEM), and cryogenic electron microscopy (cryo‐EM) images of b‐sEVs and t‐sEVs_Bone RNAs_ with enriched RNAs. SEM and TEM images of b‐sEVs and t‐sEVs_Bone RNAs_ show no difference in the morphology of sEVs, while cryo‐EM analysis suggests t‐sEVs_Bone RNAs_ contain a higher RNA content. E) RNA quantity and distribution in 1 × 10^12^ b‐sEVs, e‐sEVs_PBS_, and t‐sEVs_Bone RNAs_ (EV fraction 9–15). Synthetic mRNAs of BMP‐2 and VEGF‐A were used as standard samples (100 ng each mRNA). F) RT–qPCR analysis of BMP‐2 and VEGF‐A mRNAs indicates that t‐sEVs_Bone RNAs_ contain more transcribed BMP‐2 and VEGF‐A mRNAs than e‐sEVss_PBS_ and b‐sEVs. ^*^ *p* < 0.05 and ^****^ *p* < 0.0001. G) Schematics of a single‐sEV biochip for exosomal mRNA detection and representative total internal reflection fluorescence microscope (TIRFM) images for t‐sEVs_Bone RNAs_. Red dots: sEVs with VEGF‐A mRNAs; green dots: sEVs with BMP‐2 mRNAs; yellow dots: sEVs with both mRNAs (Scale bar: 10 µm). H) Colocalization percentage of t‐sEVs_Bone RNAs_ with both VEGF‐A and BMP‐2 mRNAs (*n* = 15). ^*^ *p* < 0.05 and ^****^ *p* < 0.0001. All data are presented as mean ± SD. Student's *t*‐test was used for comparison.

EVs released from cells are heterogeneous in size and composition because there are distinct EV subpopulations originating from different cellular pathways.^[^ ^13^ ^]^ To collect desirable therapeutic EVs after TM‐nanoEP, we sorted secreted EVs in culture medium into four different fractions by standard size exclusion chromatography (SEC) using qEV columns and characterized the distribution of therapeutic mRNAs in different EV subgroups. Most EVs in fractions 6–8 (200–650 nm) were microvesicle (MV)‐rich subpopulations (rich in MV marker: ARF6) and those in fractions 9–15 (40–250 nm) were exosome‐ or sEV‐rich subpopulations (rich in exosome or sEV markers: CD9, CD63, and TSG101) (**Figure** **2**A). Bioanalyzer assessment using synthetic mRNAs as a standard revealed that most of the full‐length transcribed BMP‐2 and VEGF‐A mRNAs (≈1500 nucleotides) were in fractions 9–15, which suggests therapeutic mRNAs are primarily encapsulated in sEV‐rich subpopulations, not MV‐rich subpopulations (Figure 2B; Figure S2A, Supporting Information). In comparison, the size distribution, morphology, zeta potential and surface markers of blank sEVs from untreated hAdMSCs (b‐sEVs) and engineered sEVs by TM‐nanoEP‐PBS treated hAdMSCs (e‐sEVs_PBS_) were similar to those of therapeutic sEVs by TM‐nanoEP‐Plasmids treated hAdMSCs (t‐sEVs _Bone RNAs_), but no large RNA was found in b‐sEVs and e‐sEVs_PBS_ (Figure 2C–E; Figure S2B, Supporting Information). Notably, both t‐sEVs _Bone RNAs_ and e‐sEVs_PBS_ contained a higher RNA amount than b‐sEVs, which suggests smaller RNAs were encapsulated in sEVs after TM‐nanoEP (Figure 2E; Figure S2B, Supporting Information). Indeed, cryo‐electron microscopy (cryo‐EM) revealed that sEVs generated by TM‐nanoEP contained many nucleic acids, whereas those from untreated hAdMSCs appeared empty (Figure 2D).

RT‐qPCR analysis showed that t‐sEVs_Bone RNAs_ contained >1000‐fold (≈1.3 copies/exosome) and >1500‐fold (≈1.8 copies/exosome) of BMP‐2 and VEGF mRNA, respectively, on average than b‐sEVs (Figure 2F; Figure S2C, Supporting Information). We then used a single‐exosome biochip assay to further characterize the t‐sEVs_Bone RNAs_, where a mixed antibody cocktail of exosome markers (CD9 and CD63) was tethered on the gold‐coated chip surface to capture individual sEVs after the purification steps. Expressions of the mRNAs of interest in the captured sEVs (BMP‐2 and VEGF‐A) were detected by MB‐FISH (molecular beacon‐based fluorescence in‐situ hybridization) probes encapsulated in cationic lipoplex nanoparticles (CLNs) and the fluorescent signal determined by a total internal reflection fluorescence microscope (TIRFM) was used to identify the mRNA content (Figure 2G; Figure S2D, Supporting Information). According to the TIRFM images (Figure 2G), >80% of the captured sEVs contained at least one transcript, and the estimated co‐localization ratio of both transcripts (BMP‐2 and VEGF‐A) within the induvial exosome was 60.57% ± 14.09% (Figure 2H).

### 2.3 Enhanced t‐sEV Release Regulated by Mechanistic Target of Rapamycin Complex 1 (mTORC1)‐Autophagy axis

**Figure 3:**
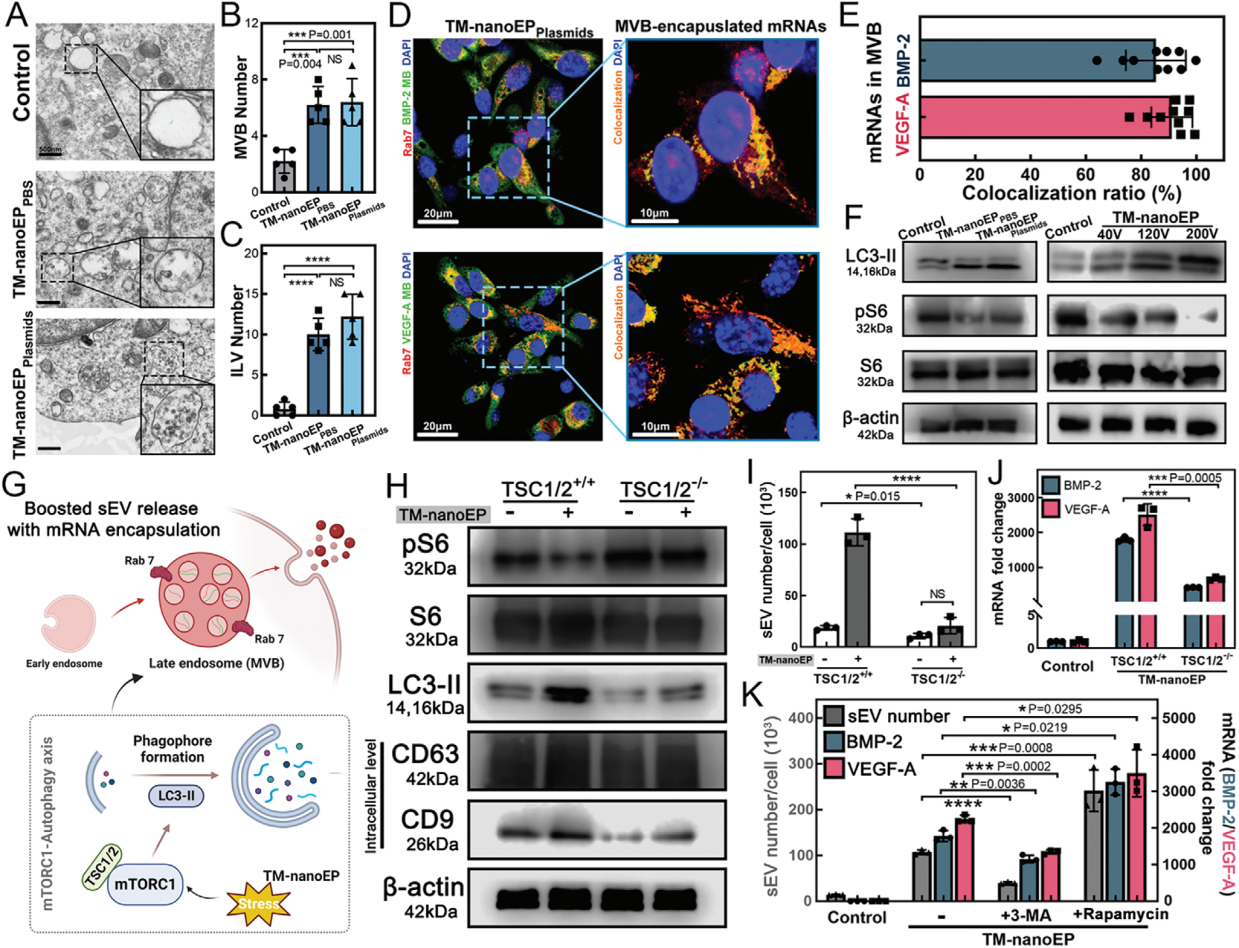
Enhanced therapeutic sEV (t‐sEV) release regulated by mTORC1‐autophagy axis. A) Cell‐electron microscopy (Cell‐EM) sections of TM‐nanoEP stimulated hAdMSCs with or without plasmids show the changes of multivesicular bodies (MVBs) and intraluminal vesicles (ILVs) versus untreated hAdMSCs as a control. Quantification of cellular cryo‐EM sections suggests TM‐nanoEP induced activity of EV precursors, B,C) MVBs and ILVs, as evidenced by a twofold increase of the MVB number and an eightfold increase of the ILV number (*n* = 5). D) mRNA cargoes (BMP‐2 and VEGF‐A) were recognized by MB‐FISH (molecular beacon‐based fluorescence in‐situ hybridization) probes (green). The late endosome/MVBs in hAdMSCs were stained by florescence‐labeled anti‐Rab7 (red). E) Colocalization quantification of mRNAs in late endosome/MVBs (*n* = 10). F) Western blot shows TM‐nanoEP suppresses mTORC1 activity (phospho‐S6 (pS6)) and thus activates autophagic activity (light chain 3‐II (LC3‐II). Increasing TM‐nanoEP voltages results in greater reduction of mTORC1 activity and enhanced autophagy. G) Illustration of mTORC1‐autophagy axis in regulating enhanced t‐sEV release by TM‐nanoEP. H) Western blot shows suppressed PS6 and enhanced LC3‐II expression with significantly increased intracellular expression sEV marker proteins (CD63 and CD9) in wide‐type hAdMSCs after TM‐nanoEP. The mTORC1 and autophagic activities in TSC1/2^−/−^ hAdMSCs were insensitive to TM‐nanoEP with no impact on sEV release. I,J) sEV number and exosomal mRNA expression by TM‐nanoEP stimulated TSC1/2^+/+^ and TSC1/2^−/−^ hAdMSCs with plasmid cocktail (*n* = 3). K) sEV number and mRNA expression by hAdMSCs pre‐treated with an mTOR inhibitor, rapamycin (400 nm), or an autophagy inhibitor, 3‐methyladenine (3‐MA, 5 mm) for 24 h before TM‐nanoEP (*n* = 3). ^*^ *p* < 0.05, ^**^ *p* < 0.01, ^***^ *p* < 0.005, ^****^ *p* < 0.0001, and ^####^ *p* < 0.0001. All data are presented as mean ± SD. Student's *t*‐test was used for comparison.

Exosomes or sEVs originate from the endosomal pathway and are formed as an intraluminal vesicle (ILV) precursors in the multivesicular body (MVB).^[^ ^14^ ^]^ Cellular cryo‐EM showed a twofold increase of MVBs and an eightfold increase of ILVs in hAdMSCs stimulated by TM‐nanoEP compared to untreated cells (**Figure** **3**A–C). Through immunofluorescence and MB‐FISH, we found that ≈85% and ≈91% of late endosome/MVBs contained BMP‐2 and VEGF‐A mRNAs, respectively, at 8 h (Figure 3D,E).

sEV release is regulated by various physiological and pathological pathways.^[^ ^15^ ^]^ Herein, we investigated how the mechanistic target of rapamycin (mTOR) complexes, specifically mTOR complex 1 (mTORC1) regulates autophagy in TM‐nanoEP for enhanced exosome secretion and vesicular mRNA loading (Figure 3G). The mTOR complex 1 (mTORC1) is sensitive to rapamycin and plays a crucial role in coordinating cellular metabolic activities in response to various external stimuli (e.g., growth factors, nutrients, and stresses).^[^ ^16^ ^]^ One major mechanism by which mTORC1 regulates cellular metabolism is through the control of autophagy for the removal of cellular organelles and components.^[^ ^17^ ^]^ Western blot analysis (Figure 3F) indicated that TM‐nanoEP negatively regulated mTORC1 and thus activated autophagic activity, as manifested by a reduction of the mTORC1 maker (phospho‐S6 (PS6)) and an increase of the autophagy marker (light chain 3‐II (LC3II), particularly at higher voltages. To further investigate the mechanism of the mTORC1‐autophagy axis in regulating exosome secretion and vesicular mRNA loading, we hyperactivated the mTORC1 expression by knocking down the tuberous sclerosis complex 1/2 (TSC1/TSC2) (Figure 3G; Figure S3A and Table S1, Supporting Information). Western blot analysis showed significantly increased intracellular expression of exosome marker proteins (CD63 and CD9) in wide‐type hAdMSCs after TM‐nanoEP. In contrast, TM‐nanoEP failed to induce any increase of exosome release in TSC2^−/−^ hAdMSCs, in which the mTORC1 and autophagy activities were largely suppressed (Figure 3H,I). Consistent with the impaired exosome release, exosomal mRNA encapsulation was also reduced in TSC2^−/−^ hAdMSCs after TM‐nanoEP when compared to the sEVs from wild‐type TSC2^+/+^ hAdMSCs (Figure 3J). To further determine the impact of the mTORC1‐autophagy axis on exosome release and mRNA encapsulation, we pre‐treated hAdMSCs with an mTOR inhibitor, rapamycin, or an autophagy inhibitor, 3‐methyladenine (3‐MA), before subjecting them to TM‐nanoEP. Rapamycin resulted in a significant increase in exosome release and mRNA loading after TM‐nanoEP, whereas 3‐MA impaired these effects (Figure 3K).

### 2.4 Efficient Translation of t‐sEVs for In Vitro Osteogenic–Angiogenic Bone Regeneration

**Figure 4:**
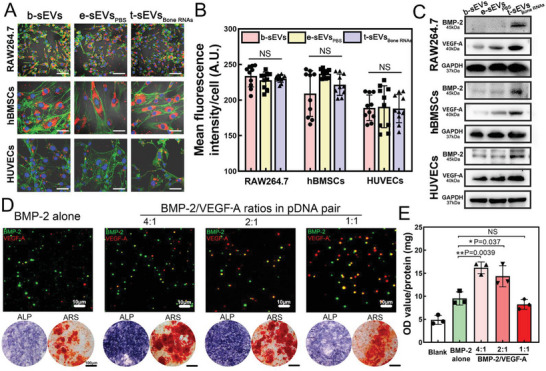
t‐sEV uptake and efficient protein translation for enhanced bone regeneration through osteogenic‐angiogenic coupling. A) Representative images of PKH26‐labeled sEVs endocytosed by RAW264.7, hBMSCs, and HUVECs after 24 h incubation. (Scale bar: 50 µm) B) Quantification of the fluorescent intensity of PKH26‐labeled sEVs in each cell. (*n* = 10) C) Translation of BMP‐2 and VEGF‐A mRNAs after t‐sEV delivery to the recipient cells for 72 h. D) Representative TIRFM images of t‐sEVs_Bone RNAs_ generated by TM‐nanoEP with different ratios of BMP‐2/VEGF‐A in plasmid cocktail, alkaline phosphatase (ALP) staining, and alizarin red staining (ARS) of hBMSCs cultured with t‐sEVs_Bone RNAs_ for 7 days. (Scale bar: 10 µm in TIRFM images and 100 µm in ALP and ARS images). E) ALP quantification in hBMSCs with different sEVs for 7 days (*n* = 3). ^*^ *p* < 0.05 and ^**^ *p* < 0.01. Student's *t*‐test was used for comparison.

In the bone healing process, monocytes/macrophages, MSCs, and endothelial cells are primary invaders at the bone defect site so they are the targets of t‐sEVs for bone regeneration.^[^ ^18^ ^]^ To investigate exosome uptake in these recipient cells, the sEVs were labeled with red PKH dye and then incubated with either murine macrophages (RAW264.7), human bone marrow mesenchymal stem cells (hBMSCs), or human umbilical vein endothelial cells (HUVECs) for fluorescence observation. Red fluorescence was observed to accumulate in the cytoplasm of all three recipient cells (**Figure** **4**A) with a similar amount of internalization (Figure 4B). There was no significant difference between the natural sEVs (b‐sEVs) and sEVs generated by TM‐nanoEP (e‐sEVs_PBS_ and t‐sEVs_Bone RNAs_). Further Western blot analysis (Figure 4C) demonstrated high mRNA translation efficiency of t‐sEVs_Bone RNAs_ in all targeted recipient cells involved in the bone repair process.

It is known that BMP and VEGF play synergistic roles in bone regeneration.^[^ ^19^ ^]^ To optimize this synergistic effect, we varied the pDNA cocktail formulation in the TM‐nanoEP process with the ratio of BMP‐2/VEGF‐A ranging from 4:1 to 1:1. The expression ratios of the two mRNAs in t‐sEVs_Bone RNAs_ characterized by the single‐exosome biochip (Figure 4D) were slightly lower than the ratios of pDNAs (Figure S4, Supporting Information). We next assessed the coupling angiogenic–osteogenic effect of t‐sEVs_Bone RNAs_ in hBMSCs using in vitro alkaline phosphatase (ALP) assay and Alizarin Red S (ARS) staining. Strong enhancement of the ALP activity and calcium deposition were found in two plasmid cocktail groups (the ratios of BMP‐2/VEGF‐A pDNAs were 4:1 and 2:1), while a 1:1 ratio of BMP‐2/VEGF‐A pDNAs or BMP‐2 pDNA alone did not perform as well (Figure 4D,E).

### 2.5 Synergistic Therapeutic Effects of Exosomal microRNAs (miRNAs) Induced by Bone‐Coupling mRNAs in t‐sEVs

**Figure 5:**
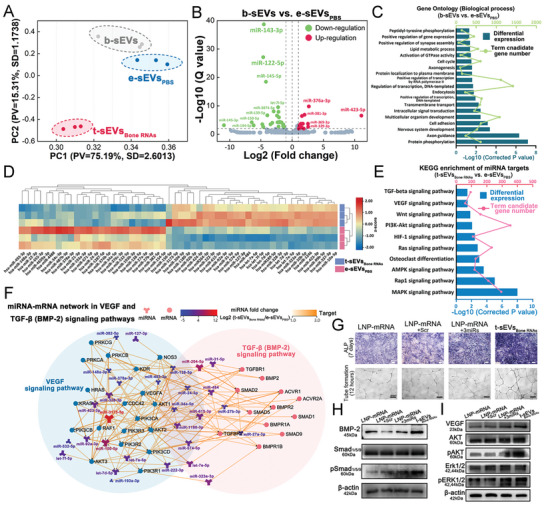
Profiling and synergistic therapeutic effects of microRNAs induced by bone‐coupling mRNAs in sEVs. A) Principal component analysis (PCA) of microRNAs of sEVs from different conditions. B) Volcanoplot of the differentially expressed microRNAs of sEVs from hAdMSCs with and without TM‐nanoEP (e‐sEVs_PBS_ vs b‐sEVs). C) Gene Ontology (GO) analysis of the biological process (e‐sEVs_PBS_ versus b‐sEVs). *q* value < 0.05. D) Heat map of the differentially expressed microRNAs in sEVs from hAdMSCs under TM‐nanoEP‐PBS and TM‐nanoEP‐Plasmids (t‐sEVs_Bone RNAs_ versus e‐sEVs_PBS_). E) Top KEGG pathway enrichment of signal transduction and development, and regeneration pathways related to bone regeneration (t‐sEVs_Bone RNAs_ vs e‐sEVs_PBS_). *p* value < 0.05. F) Interaction network of TGF‐β (BMP‐2) and VEGF signaling‐related microRNAs/target mRNAs. G) Alkaline phosphatase (ALP) staining (upper row) of hBMSCs and tube formation of HUVECs (bottom row) cultured with LNP‐mRNA, LNP‐mRNA+Scr, LNP‐mRNA+3miRs, and t‐sEVs_Bone RNAs_ for 7 days and 12 h (scale bar: 100 µm). Western blot analysis of expression of H) BMP‐2 and I) VEGF and their downstream signaling pathways.

Some recent studies showed that abundant microRNAs (miRNAs) encapsulated in sEVs may play a regulatory role and can be a promising therapeutic factor in bone regeneration.^[^ ^20^ ^]^ To better understand the osteogenic and angiogenic capacities of exosomal miRNAs from hAdMSCs stimulated by TM‐nanoEP with or without the bone plasmid cocktail, we conducted small RNA‐seq and analyzed the differences between cohorts using Gene Ontology (GO) and Kyoto Encyclopedia of Genes and Genomes (KEGG) Enrichment analyses. Duplicate plots within the three sEV groups by principal component analysis (PCA) (**Figure** **5**A) revealed clusters, indicating the stability of the component and notable differences between the cohorts. More specifically, the e‐sEVs_PBS_ cluster was closely aligned with the b‐sEVs cluster across both primary PC axes, indicating similar miRNA composition after TM‐nanoEP treatment with only PBS. On the other hand, the t‐sEVs_Bone RNAs_ cluster was distinctly positioned away from both the e‐sEVs_PBS_ and b‐sEVs clusters on both PC axes. This distinction implied that plasmid transfection induced a more profound effect on the change of exosomal miRNA composition when compared to the TM‐nanoEP process. When comparing b‐sEV and e‐sEV_PBS_ on the component discreteness, a total of 45 differentially expressed miRNAs (19 down‐regulated and 26 up‐regulated) were detected in the volcanoplot (Figure 5B; Table S2, Supporting Information). Target genes of these differentially expressed miRNAs with the top 20 significant biological processes (Figure 5C), molecular functions (Figure S5A, Supporting Information), and cellular components (Figure S5B, Supporting Information) were identified.

We then analyzed the differences of miRNAs in e‐sEV_PBS_ and t‐sEVs_Bone RNAs_ for target gene functions by GO and KEGG analyses. A total of 52 differentially expressed miRNAs (24 down‐regulated and 28 up‐regulated) were detected and plotted as a heatmap in Figure 5D and Table S3 (Supporting Information). Target genes of these differentially expressed miRNAs were further predicted and connected through KEGG pathway analysis (Figure 5E). The prediction showed enrichment of signal transduction and development and regenerative pathways related to bone regeneration, including central pathways for cellular programs (MAPK signaling pathway, AMPK signaling Pathway, PI3K‐Akt signaling pathway, and RAS signaling pathway), developmental signaling pathways (Wnt signaling pathway and osteoclast differentiation), cellular response pathway (HIF‐1 signaling pathway), and two signaling pathways (TGF‐β (58 targeted genes) and VEGF (91 targeted genes)) directly associated with the introduced bone plasmid cocktail, BMP‐2 and VEGF‐A. Both VEGF and TGF‐β signaling pathways exhibited significant enrichment (P value < 0.05) when comparing the sEVs from hAdMSCs with and without plasmid cocktail transfection (Table S4, Supporting Information). In comparison, only the VEGF signaling pathway, which is intrinsically enriched in hAdMSC‐derived sEVs, showed a difference between b‐sEVs and e‐sEVs_PBS_. (Table S5, Supporting Information).

Network interactions between miRNAs and their targeted mRNAs in TGF‐β and VEGF signaling pathways (Figure 5F) were then constructed to further investigate the therapeutic effects of miRNAs induced by bone‐coupling mRNAs in t‐sEVs. We identified 29 miRNAs that were differentially expressed and highly associated with genes involved in TGF‐β and VEGF signaling pathways (Figure 5F; Table S6, Supporting Information). Based on the fold change, expression level, and targeted gene nodes within the two pathways, the top 10 out of the 29 miRNAs were selected for further validation of their osteogenic‐angiogenic effects (Table S6, Supporting Information). RT‐qPCR analysis further demonstrated that three of these miRNAs, let‐7a‐5p, let‐7e‐5p, and miR‐92a‐3p, exhibited the highest fold change in the expression of associated osteogenic markers (ALP, BMP‐2, and RUNX2) and angiogenic markers (VEGF, PECAM, and eNOS) (Figure S5C, Supporting Information). As a result, these three miRNAs were selected for evaluation of their synergistic effects on miRNA‐mRNA interactions. In vitro ALP and tube formation assays showed higher osteogenic and angiogenic activities of t‐sEVs_Bone RNAs_ (1 × 10^11^ particles) when compared to the lipid nanoparticles (LNPs) with the same synthetic mRNA amount encapsulated (LNP‐mRNAs with 100 ng BMP‐2 mRNA and 30 ng VEGF‐A mRNA incorporated). Notably, adding the three miRNA mimics (let‐7a‐5p, let‐7e‐5p, and miR‐92a‐3p) into LNP‐mRNAs (LNP‐mRNAs+3miRs) improved the tube formation ability to a level comparable to that of t‐sEVs_Bone RNAs_. However, the ALP activity of LNP‐mRNAs+3miRs was still lower than that of t‐sEVs_Bone RNAs_ (Figure 5G; Figure S5D,E, Supporting Information). Western blot analysis (Figure 5H,I) showed higher BMP‐2 and VEGF protein expressions in hBMSCs and HUVECs treated with t‐sEVs_Bone RNAs_ than in LNP‐mRNAs. Adding the three miRNAs mimics significantly enhanced the expression of BMP‐2 and VEGF proteins as well as their downstream pathway proteins (pSmad1/5/9, pERK, and pAKT) in the recipient cells. However, t‐sEVs_Bone RNAs_ still provided better osteogenic‐angiogenic activities than LNP‐mRNAs+3miRs. These results indicate that t‐sEVs_Bone RNAs_ produced from hAdMSCs by TM‐nanoEP contain enriched miRNAs synergized with the transcribed therapeutic mRNAs and thus render more favorable osteogenic‐angiogenic efficacy compared to the conventional LNP‐mRNA systems. It should be noticed that sEVs are also rich in biomolecules other than miRNAs. Changes in protein and small molecule contents in t‐sEVs may also affect the osteogenic‐angiogenic efficacy. This needs to be further investigated.

### 2.6 A Customized Hydrogel Cage Loaded with RNA‐Enriched t‐sEVs in In Vitro Bone Tissue Regeneration

**Figure 6:**
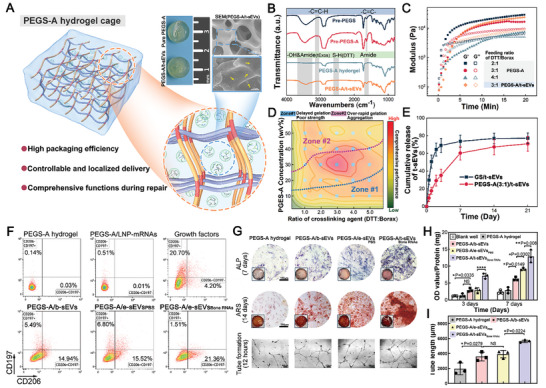
Design of customized PEGylated poly (glycerol sebacate) acrylate (PEGS‐A) hydrogel cage with RNA‐enriched t‐sEVs and in vitro demonstration of osteogenic‐angiogenic coupling for bone tissue regeneration. A) Schematic illustration of PEGS‐A hydrogel cage with properties that enable rapid assembly and good distribution of t‐sEVs, efficient local delivery of therapeutic RNAs, desirable injectability, mechanical support, and enhanced regenerative capacity. B) FT‐IR spectra of PEGS pre‐polymer, PEGS‐A hydrogel, and PEGS‐A/t‐sEVs. Sharp and broad absorption bands ≈1750 and 3500 cm^−1^ indicate stretching vibration of carbonyl groups and free hydroxyl groups (O─H) or amide groups (N─H). Amide groups (C═O stretching) from incorporated sEVs also can be found at ≈1660 cm^−1^. Peaks of Alkenyl C─H and C═C stretching are located at ≈3050 and 1640 cm^−1^. The absence of S─H stretching (≈2600 cm^−1^) indicates the gelation of PGES‐A pre‐polymer. C) Rheological behaviors of the PEGS‐A pre‐polymers with different ratios of Borax/DTT. D) Orthogonal analyses of PEGS‐A formulations for bone tissue engineering. Zone #1 indicates the hydrogel with delayed gelation and poor strength, while Zone #2 indicates the hydrogel with too fast gelation and the occurrence of sEV aggregation. E) Release profiles of t‐sEVs from gelatin sponge (GS) and PEGS‐A hydrogel. F) Percentages of M1 and M2 polarization of RAW 264.7 co‐cultured with pure hydrogel, LNPs/synthetic mRNAs, growth factors (rhVEGF‐A and rhBMP‐2), PEGS‐A/b‐sEVs, PEGS‐A/e‐sEVs_PBS_, and PEGS‐A t‐sEVs_Bone RNAs_ for 72 h. G) Alkaline phosphatase (ALP) staining (upper row) and Alizarin red staining (ARS) (second row) of rBMSCs, and tube formation of HUVECs cultured with pure hydrogel, PEGS‐A/b‐sEVs, PEGS‐A/e‐sEVs_PBS_, and PEGS‐A t‐sEVs_Bone RNAs_ for 7, 14 days, and 12 h (scale bar: 100 µm). Quantitative analyses of H) ALP activity of rBMSCs and I) tube formation of HUVECs on the PEGS‐A/sEVs hydrogels for 3 and 7 days, and 12 h (*n* = 3). ^*^ *p* < 0.05, ^**^ *p* < 0.01, and ^****^ *p* < 0.0001. All data are presented as mean ± SD. One‐way ANOVA was used for comparison.

EVs have been explored in tissue engineering, but their tissue retention is poor, and long‐term functions are often insufficient for regeneration.^[^ ^21^ ^]^ Although a few hydrogels have been used to pack growth factors or small therapeutics for tissue regeneration, they are not ideal for sEVs carrying genetic cargoes because hydrogel formation requires external gelation techniques such as ultraviolet (UV) light and thermal processes, which are detrimental to sensitive RNAs in sEVs.^[^ ^22^ ^]^ Herein, we customized a PEGylated poly (glycerol sebacate) acrylate (PEGS‐A) injectable hydrogel cage, which can rapidly immobilize in vivo at body temperature and can controllably release sEVs through surface erosion. These characteristics are essential for therapeutic genetic materials delivered by sEVs to fulfill regeneration requirements in bone repair. First, PEGS‐A pre‐polymers were prepared using an acylation reaction from PEGS pre‐polymers (Figure S6A, Supporting Information). To secure desirable hydrogel features (e.g., high water content) and functional hydroxyl group for crosslinking, the molar ratio of PEG to glycerol in PEGS pre‐polymer was set as 30%.^[^ ^23^ ^]^ The successful synthesis of the PEGS pre‐polymer and the PEGS‐A pre‐polymer was confirmed by Fourier‐transform infrared spectroscopy (FT‐IR) and proton nuclear magnetic resonance (^1^H NMR). Both PEGS and PEGS‐A pre‐polymers exhibited sharp and broad absorption bands ≈1750 and 3500 cm^−1^, which correspond to the stretching vibration of carbonyl groups, and free hydroxyl groups or amide groups, respectively (**Figure** **6**B). PEGS‐A pre‐polymer showed a weak peak ≈3050 cm^−1^ that belongs to the ─C═C─H of the grafted acryloyl groups. The graft of acryloyl groups in PEGS‐A was also confirmed by the occurrence of new peaks assigned to the methylene and methine groups of acrylic segments at 5.83 and 6.42 ppm (peaks g and i) and 6.12 ppm (peak h), respectively (Figure S6B, Supporting Information).

The PEGS‐A hydrogel was then formed by the thiol‐Michael addition click reaction between acrylate and dithiothreitol (DTT) under the catalysis of Borax (Figure S6A, Supporting Information). Unlike other exosome delivery systems, t‐sEVs can be directly involved in the one‐pot synthesis without risks of membrane disruption and therapeutic RNA degradation as the reaction proceeds rapidly in aqueous media at a low temperature (4 °C).^[^ ^20^, ^24^ ^]^ According to the FT‐IR spectrum in Figure 6B, the PEGS‐A hydrogel with sEVs showed much stronger peaks of amine and hydroxyl groups than the hydrogel only, suggesting successful encapsulation of sEVs after gelation. This can be further confirmed in scanning electron microscopy (SEM) images with observable convex vesicles on the hydrogel surface (Figure 6A; Figure S6E, Supporting Information). To improve the performance of PEGS‐A/sEVs hydrogel in bone repair, the PEGS‐A pre‐polymer concentration and the ratio of Borax and DTT were investigated through orthogonal analyses. The pre‐polymer concentration was varied in the range of 10–50% to evaluate injectability and gelation ability. Among them, 10%, 40%, and 50% led to either a prolonged gelation time or too high a viscosity (fast crosslinking) causing exosome aggregation (Figure S6C,E, Supporting Information). Therefore, the 20–30% of PEGS‐A pre‐polymer concentration was further studied for desirable exosome distribution and gelation time. We selected 30% because this concentration provided higher mechanical strength (Figure S6D and Video S1, Supporting Information). With the polymer concentration set at 30%, the molar ratio of DTT and Borax was then varied from 5:1 to 1:1 to evaluate the rheological behavior of the pre‐polymers during gelation. A DTT/Borax molar ratio of 5:1 was too high to form a gel and a ratio of 1:1 would lead to uncontrollable crosslink, while ratios between 4:1 and 2:1 could provide good injectability and gelation ability. Dynamic moduli of the pre‐polymers with time sweeping were tested to determine the time‐dependent rheological performance. All the ratio groups ranging from 4:1 to 2:1 showed viscous characteristics (*G*′<*G*″) first, and gradually exhibited elastic behaviors, which indicated a gelling transition as confirmed by the higher G’ (Figure 6C). As expected, the hydrogel processor with the highest Borax proportion (2:1) led to rapid crosslinking within 1 min thereby causing a poor exosome distribution. By contrast, ratios of DTT/Borax at 3:1 and 2:1 showed a significantly prolonged state of viscous fluid before the crosslinking point (*G*′ = *G*″, ≈3 and 10 min, respectively). Adding sEVs in pre‐polymers did not affect the gel formation characteristics. From these orthogonal analyses (Figure 6D), PEGS‐A with a 30% pre‐polymer concentration and a ratio of DTT/Borax at 3:1 in the precursors was considered as the optimum formulation for t‐sEVs delivery in bone regeneration. Compared to conventional exosome loading systems like gelatin sponge (GS), the PEGS‐A hydrogel allowed extended retention and controllable release of sEVs (Figure 6E), which is essential for localized in vivo delivery of genetic cargoes.

In the initial stage of bone healing, macrophages (*M*_φs_) are activated to M1 *M*_φs_ (pro‐inflammation) which, however, would impair fracture healing in the later stage.^[^ ^25^ ^]^ This is the reason that mesenchymal stem cells (MSCs)‐derived sEVs have been highly appreciated in many disease treatments, including bone regeneration because of their anti‐inflammatory effects (induce M2‐like *M*_φs_) through M1/M2 *M*_φs_ regulation.^[^ ^26^ ^]^ To explore the impact of immunomodulation on bone repair, we compared pure hydrogel, PEGS‐A/b‐sEVs, PEGS‐A/e‐sEVs_PBS_ and PEGS‐A/t‐sEVs_Bone RNAs_ against conventional growth factors (1 µg rhBMP‐2 and 0.3 µg rhVEGF‐A) and LNPs/synthetic mRNAs (100 ng BMP‐2 mRNAs and 30 ng VEGF mRNAs) by co‐culturing them with a macrophage cell line, RAW 264.7 for phenotype transition. Slight pro‐inflammation was found in pure hydrogel (0.14% M1 phenotype) and LNPs/ synthetic mRNAs (0.51% M1 phenotype) cohorts. Notably, significant M1‐like polarization (20.07% M1 phenotype) was found in the growth factors cohort (Figure 6F), which partially contributes to the severe side effects in conventional growth factor‐based therapies. In contrast, all PEGS‐A/sEVs hydrogels showed remarkable Mφs polarization toward the M2 phenotype (14.94%, 15.52%, and 21.36% M2 phenotype in PEGS‐A/b‐sEVs, PEGS‐A/e‐sEVs_PBS_, and PEGS‐A/t‐sEVs_Bone RNAs_, respectively), and the PEGS‐A/t‐sEVs_Bone RNAs_ provided the highest M2 Mφs polarization, which could be ascribed to the bone‐related mRNAs and their induced miRNAs in sEVs. ELISA tests of cytokines secreted by Mφs after lipopolysaccharide (LPS) stimulation (Figure S6F–H, Supporting Information) showed that exosome treatment could significantly reduce the LPS‐stimulated levels of TNF‐α and IL‐6 (M1 markers), and increase the expression level of IL‐10 (M2 maker), particularly in the t‐sEVs_Bone RNAs_ cohort. These results further supported the superior anti‐inflammatory abilities of PEGS‐A/t‐sEVs_Bone RNAs_.

We next assessed the coupling angiogenic–osteogenic effect of the PEGS‐A/sEVs hydrogel in bone regeneration using in vitro alkaline phosphatase (ALP) assay, Alizarin Red S (ARS) staining, and tube formation. All exosome groups exhibited enhanced 7‐day ALP activity when compared to the pure hydrogel, and the most intensive ALP expression was observed in PEGS‐A/t‐sEVs_Bone RNAs_ (Figure 6G). Quantitative analysis of the ALP activity on both days 3 and 7 also showed a trend similar to the staining results (Figure 6H). Notably, there was a significant difference in the ALP activity between PEGS‐A/b‐sEVs and PEGS‐A/e‐sEVs_PBS_ on day 7, implying that the increased miRNA cargo induced by TM‐nanoEP might contribute to osteogenesis. The results of ARS exhibited a similar trend (Figure 6G) that mineral deposition on PEGS‐A/t‐sEVs_Bone RNAs_ was the most prominent group. Tube formation of HUVECs further demonstrated the highest angiogenic capacity of PEGS‐A/t‐sEVs_Bone RNAs_ (Figure 6I). Together, the PEGS‐A hydrogel cage can pack t‐sEVs, allowing efficient translation of RNA therapeutics mediated by t‐sEVs for coupled angiogenesis‐osteogenesis to meet comprehensive requirements in bone regeneration.

### 2.7 In Vivo Demonstration of Bone RNA‐Enriched sEVs within Injectable Hydrogel Cage in Rats with Critical‐Size Femoral Defects

**Figure 7:**
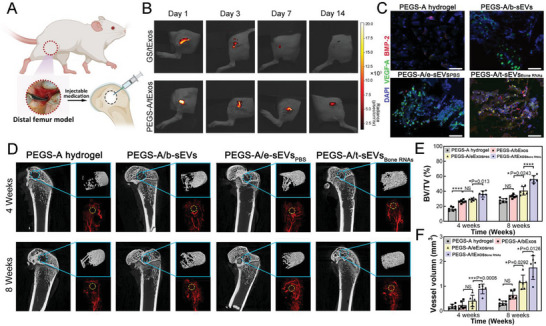
In vivo regenerative efficacy of therapeutic PEGS‐A/sEVs hydrogel. A) Schematic illustration of injection treatment in rats with a critical‐size femoral defect. B) In vivo fluorescence imaging of rats with implantation of gelatin sponge (GS) or injection PEGS‐A hydrogel containing PKH26‐labelled sEVs on 1st, 3rd, 7th, and 14th day post‐delivery (*n* = 6 in each cohort). C) Fluorescence images of cells within the pure hydrogel, PEGS‐A/b‐sEVs, PEGS‐A/e‐sEVs_PBS_, and PEGS‐A/t‐sEVs_Bone RNAs_ immunostained with VEGF‐A (green) and BMP‐2 (red) on 7th day after injection. D–F) Micro‐CT images obtained from the reconstruction of the femur defects and illustration of new bone and vessel formation in the 4th and 8th week. Morphometric analyses of bone volume/total volume (BV/TV) and vessel volume for pure PEGS‐A hydrogel and PEGS‐A/sEVs cohorts after 4 and 8 weeks of treatment (*n* = 6 for each cohort). ^*^ *p* < 0.05, ^***^ *p* < 0.005, and ^****^ *p* < 0.0001. All data are presented as mean ± SD. One‐way ANOVA was used for comparison.

After in vitro demonstration, we tested the in vivo angiogenesis‐osteogenesis of the PEGS‐A/t‐sEVs in rats with critical‐sized femoral defects (**Figure** **7**A). For in vivo delivery, we immobilized 1 × 10^12^ sEVs within each hydrogel, incorporating ≈1 µg of BMP‐2 mRNA and 0.3 µg of VEGF mRNA specifically within the PEGS‐A/t‐sEVs_Bone RNAs_ cohort. To assess whether the PEGS‐A hydrogel cage improved the in vivo distribution and translation of therapeutics mediated by the sEVs, we first performed in vivo fluorescence imaging of PKH26‐labelled sEVs within injected PGESA hydrogel or implanted GS in defect sites over 14 days. As shown in Figure 7B and Figure S7A (Supporting Information), exosome delivery was confirmed by strong in vivo PKH26 signals, and PGES‐A hydrogel showed a significantly higher fluorescence signal than GS (82.90 ± 12.11% vs 44.59 ± 11.19%, ^***^ *p* = 0.002) after 3 days. The exosome signal could be observed in PEGS‐A hydrogel on day 7 and day 14 (58.06 ± 5.20% and 23.30 ± 9.53%), while the signal in the GS scaffold has largely decayed (9.25 ± 13.45% and 3.14 ± 2.42%) during the same time period. Further tissue distribution after 14 days showed that sEVs encapsulated in PEGS‐A hydrogel were locally and slowly delivered, leading to lower hepatic, splenic, and renal accumulation than those immobilized in GS (Figure S7A, Supporting Information). We then assessed the localized translation of genetic materials mediated by sEVs within the injected PEGS‐A hydrogel and all PGES‐A/sEVs hydrogels showed good cell infiltration on day 7 (Figure 7C). Host cells within the PEGS‐A/t‐sEVs_Bone RNAs_ exhibited high expressions of BMP‐2 (red) and VEGF (green), indicating a high translation efficacy of the exosomal mRNAs carried by the t‐sEVs_Bone RNAs_.

We next tested whether the utilization of a PEGS‐A/sEVs hydrogel delivery system carrying angiogenetic‐osteogenic mRNAs could result in improved bone regeneration in rats with critical‐size femoral defects. In the 4th and 8th week post‐injection, high‐resolution micro‐CT scanning was performed to observe and quantify the regenerated bone and vessels. With the injection of PEGS‐A/sEVs hydrogels, the new bone and vessel covered the defects concavely in the 4th week, and the newly formed tissues were integrated with the surrounding tissues after 8 weeks (Figure 7D). In comparison, the matrix degraded over time in the pure hydrogel cohort, and there was no apparent formation of bone or vessel in the center of the defect in the 8th week. The morphometrical analyses (Figure 7E,F; Figure S7C,D, Supporting Information) further confirmed a significantly higher percentage of regenerative parameters in PEGS‐A/sEVs hydrogel cohorts compared with the pure hydrogel cohort, including the relative/tissue volume (BV/TV), vessel volume, bone mineral density (BMD) and trabecular thickness (Tb.Th.). A significant difference of BV/TV, Tb.Th., and vessel volume could be found between PEGS‐A/b‐sEVs and PEGS‐A/e‐sEVs_PBS_ in the 8th week, implying that the increased miRNAs in sEVs induced by TM‐nanoEP with only PBS may contribute to in vivo bone regeneration. Again, PEGS‐A/t‐sEVs_Bone RNAs_ performed the best for all regenerative parameters at different time points when compared with other exosome cohorts, indicating efficient bone regeneration based on angiogenic–osteogenic coupling functions from enriched mRNAs and miRNAs within the sEVs.

**Figure 8:**
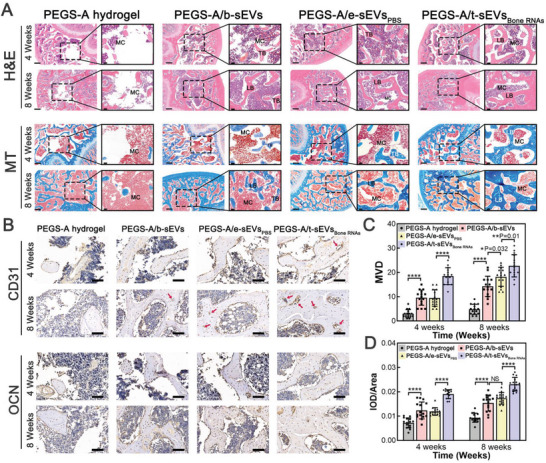
Histological and immunohistochemical analyses of bone tissue regeneration in rats with distal femur critical‐size defects in the 4th and 8th week after injection of PEGS‐A/sEVs hydrogels. A) Histological evaluation of regenerative bone sections by H&E and Masson's trichrome (MT) staining in the 4th and 8th week after injection. The black frame presents high‐resolution images of defect sites. TB: trabecular bone; LB: lamellar bone; MC: marrow cavity. (Scale bar: 500 and 100 µm for low‐power (2x) and high‐power (10x) objectives, respectively). B) Immunohistochemical staining for CD31 and OCN in the 4th and 8th week after injection. Red arrows indicate new blood vessel formation. (Scale bar: 50 µm). Quantitative analysis of C) microvessel density (MVD) and D) integral optical density (IOD)/area of positive signals (*n* = 15, three areas of five slice replicates in each cohort). ^*^ *p* < 0.05, ^**^ *p* < 0.01, and ^****^ *p* < 0.0001. All data are presented as mean ± SD. One‐way ANOVA was used for comparison.

Histological analyses, including hematoxylin and eosin (H&E) staining and Masson's trichrome (MT) staining performed in the 4th and 8th week, confirmed the osteogenic and angiogenic capacities of injected PEGS‐A/sEVs hydrogels (**Figure** **8**A). The defect site with pure hydrogel injection was primarily filled with marrow cavities caused by material degradation, while a lot of newly formed trabecular bones or lamellar bones could be observed in the defects with injected PEGS‐A/sEVs hydrogels. After both time points, contiguous formation of mature lamellar bones could be identified in PEGS‐A/t‐sEVs_Bone RNAs_, suggesting a high regenerative efficiency by delivering enriched angiogenic–osteogenic RNAs within the sEVs. IHC staining of CD31 and OCN expressions around the defect tissues further confirmed the quantity and quality of newly formed vessels and bones in the femur head defects (Figure 8B). Of all cohorts, PEGS‐A/t‐sEVs_Bone RNAs_ again showed the most abundant invaded vessels and OCN‐positive areas in both the 4th and 8th week post‐injection (Figure 8C,D). Taken together, these findings demonstrate that TM‐nanoEP‐induced t‐sEVs with enriched angiogenic–osteogenic mRNAs and associated miRNAs delivered by a new injectable PEGS‐A hydrogel cage are able to rapidly heal critical‐sized bone defects with high regenerative quality in a rat model.

## 3 Discussion

The use of extracellular vesicles (EVs) as a therapeutical system is gaining popularity in the field of regenerative medicine due to their biocompatibility, low immunogenicity, and cell‐free features. However, current EV therapy faces multiple challenges, including limited therapeutic RNA cargo loading, low production from parental cells, and very short in vivo shelf life. Our study presents a clinically viable and scalable method by employing cellular nanoelectroporation with commercially accessible track‐etched membranes (TM‐nanoEP) for generating substantial quantities of small EVs (sEVs) containing therapeutic mRNAs and associated microRNAs (miRNAs). Unlike traditional post‐loading of therapeutics into EVs, our technique utilizes selected parental cells as a bioreactor to endogenously generate therapeutic mRNAs through the transcription of plasmid DNAs (pDNAs), load them in MVBs/ILVs (precursor of sEVs), and release therapeutic sEVs (t‐sEVs) into the culture medium via exocytic secretion.^[^ ^27^ ^]^ Through the optimization of electroporation conditions and EV collection (for hAdMSCs: 120 V, 10 pulses, 0.1s interval for cell poration and 16 h for EV collection), we demonstrate that TM‐nanoEP allows high loading of multiple functional mRNAs (BMP‐2 and VEGF‐A) in abundant sEVs. This breakthrough dramatically enhances the efficacy of current EV therapies based on naturally released or exogenously loaded small RNAs (e.g., miRNAs and siRNA/shRNA). Delivering pDNAs to the parental cells not only leads to the production of desired mRNAs but also enhances the expression of miRNAs associated with the pDNAs in secreted sEVs. This mRNA‐miRNA combination provides a synergistic impact on EV efficacy. By incorporating BMP‐2 and VEGF‐A mRNAs (a pDNA ratio of 4:1 for a mRNA ratio of ≈3:1) inside sEVs, we demonstrate potent bone regeneration that couples osteogenesis and angiogenesis in a challenging critical‐size defect model.

After TM‐nanoEP, cells are observed to maintain homeostasis by negatively regulating the mammalian target of Rapamycin complex 1 (mTORC1) and subsequently inducing autophagy activation, which is essential for cell survival toward stresses. With the knockdown of the tuberous sclerosis complexes (TSC) upstream and mTORC1 activation, both exosome release and mRNA expression decrease, suggesting the involvement of the mTOR‐autophagy axis in the TM‐nanoEP process. Pre‐treating parental cells with Rapamycin and/or an inhibitor of mTOC1 can further boost exosome production and the encapsulation of therapeutic mRNAs in TM‐nanoEP, presenting a highly encouraging combination strategy for increasing the yield of t‐sEVs with enriched mRNA loading. It should be noticed that other intracellular pathways could be involved in exosome or sEV biogenesis and cargo sorting resulting from external stimuli, such as ESCRT machinery, calcium‐dependent exocytosis, and impaired lysosomal activities. More specifically, when parental cells respond to TM‐nanoEP stimuli, to maintain cellular fitness, the dynamics of sEV release, cargo sorting, autophagy, and lysosomal activities, as well as their interplay, can differ from those observed in unstressed conditions.^[^ ^16^, ^28^ ^]^ All of these factors need further investigation to provide a better understanding of the mechanisms underlying t‐sEVs generated by TM‐nanoEP.^[^ ^29^ ^]^

To adapt our therapeutics sEVs for bone regeneration, we further incorporated a customized PEGS‐A injectable hydrogel cage to facilitate localized administration of the sEVs with enriched RNAs, resulting in more favorable repair outcomes. While a few hydrogels have been used to pack therapeutics or EVs for enhanced retention in tissue regeneration,^[^ ^22^, ^24^, ^30^ ^]^ their synthesis procedures are not ideal for our t‐sEVs carrying sensitive genetic cargoes. The primary reason is that most current hydrogel formation methods require specific organic solution phases and external gelation techniques such as ultraviolet (UV) light and thermal processes, which can be detrimental to sensitive mRNAs present in our t‐sEVs.^[^ ^22^ ^]^ In contrast, the PEGS‐A hydrogel in this study can self‐shape in a water‐based phase through one‐pot thiol‐Michael addition at a low temperature (4 °C), without requiring external crosslinking stimuli (Figure 6). These exceptional characteristics ensure the preservation of t‐sEVs integrity and sensitive mRNAs incorporated prior to implantation, thereby maximizing the translational efficacy of the genetic materials they contain. Furthermore, the PEGS‐A with customized modulation over pre‐polymers and crosslinking agents contributes to the extended retention of therapeutic exosomes in the defect sites and promotes their sustained functionality for effective regeneration. With this injectable PEGS‐A hydrogel cage that packed t‐sEVs enriched with angiogenic–osteogenic RNAs, healing of critical‐sized bone defects is expedited with exceptional regenerative quality. It is also worth noting that e‐sEVs_PBS_ generated by TM‐nanoEP with PBS exhibits an enhanced ossification compared to the native b‐sEVs. The observed improvement can be attributed to the inherent therapeutic potential of the parental cells used in this work, and TM‐nanoEP, to some extent, amplifies this therapeutic potential by increasing the miRNA content and introducing other factors (e.g., heat shock proteins (HSPs)) (Figure S8, Supporting Information) in the sEVs.^[^ ^31^ ^]^ Further studies are required to verify if this phenomenon is applicable in other regenerative areas.

Currently, the TM‐nanoEP cycle includes three phases: cell spreading (16–18 h), EV generation (16–20 h), and EV purification and isolation (1–2 h). In total, the entire process spans ≈2 days. By utilizing a 100 cm^2^ track‐etched PET membrane with ≈10^7^ AdMSCs, a single TM‐nanoEP cycle can generate ≈10^12^ therapeutic sEVs (t‐sEVs) which carry ≈1 µg BMP‐2 and 0.3 µg VEGF‐A mRNAs. This quantity proves sufficient to yield favorable restorative outcomes in a preclinical in vivo rat model featuring a critical‐size defect of Ø 3 mm × 5 mm (Figures 7 and 8). However, for the clinical translation potential of TM‐nanoEP‐induced t‐sEVs toward human bone regeneration, such yield is insufficient for human clinical, particularly one of the most formidable scenarios: the large segmental defect. Typically, a human segmental defect can extend beyond 6 cm in length and possess a diameter of roughly 2 cm. This volumetric size surpasses that of the rat preclinical model employed in this manuscript by over 500 folds.^[^ ^32^ ^]^ To effectively address such a substantial human clinical‐scale defect, an estimated 10^15^ t‐sEVs would be necessary because the argumentation in therapeutic dosage for bone regeneration must surpass the growth in the volumetric size of the defect. Generating this quantity would entail utilizing ≈10^10^ AdMSCs in a single TM‐nanoEP cycle. Given that track‐etched membranes are both readily accessible and cost‐effective (around US$30 for a 4A‐sized sheet (20 cm × 30 cm), sourced from AR Brown‐US, LLC), the mass production of sEVs for clinical use can be achieved in a cost‐effective manner. For ≈10^10^ AdMSCs in the TM‐nanoEP process comprising five reusable cycles, ≈35 4A‐sized tack‐etched membrane sheets are required for a total cost of about US$1000.

It is worth noting that these estimates could considerably fluctuate based on the nature and dimensions of the defect or fracture. Determining the optimal dosage of t‐EVs for human patients should involve extensive monitoring over an extended time period and the observation of regenerative outcomes in clinical trials. Irrespective of the specific scenarios, it remains essential to design high‐throughput cell transfection techniques and scale‐up sEV production processes. Within our manuscript, we have discovered the pivot role of the mTORC1‐autophagy axis in instigating therapeutic EVs through TM‐nanoEP (Figure 3). By pre‐treating parental cells with Rapamycin or other inhibitors/enhancers of mTORC1 (Figure 3), we can amplify sEVs production and agument the encapsulation of therapeutic mRNAs subsequent to TM‐nanoEP. This combination approach exhibits promising potential for further increasing the yield of t‐sEVs with high mRNA loading. Furthermore, we are actively engaged in the development of microfluidics‐enhanced TM‐nanoEP processes to reduce the cycle time and to further increase the sEV/mRNA production per cell. In summary, we believe that TM‐nanoEP‐induced t‐sEVs hold promise not only for future bone regeneration therapies but also as a novel way of treating other diseases.

## 4 Experimental Section

### Cell Culture and Plasmids Preparation

Human adipose‐derived mesenchymal stem cells (hAdMSCs, PCS‐500‐011) and human bone (hBMSCs, PCS‐500‐012) purchased from American Type Culture Collection (ATCC) were cultured in mesenchymal stem cell basal medium (PCS‐500‐030, ATCC) supplemented with mesenchymal stem cell growth kit (PCS‐500‐040, ATCC) and 1% penicillin–streptomycin at 37 °C in humidified conditions equilibrated with 5% CO_2_. Rat bone marrow stromal cells (rBMSCs) were extracted from the femur bone marrow of 4 weeks old male SD rats. Primary rBMSCs were expanded in α‐MEM (Gibco) containing 10% fetal bovine serum (FBS, Gibco) and 1% penicillin–streptomycin. Murine macrophages (RAW 264.7) and human umbilical vein endothelial cells (HUVECs) were purchased from Shanghai Institutes for Biological Sciences (SIBS, Shanghai, China) and grown in DMEM (Gibco) supplemented with 10% FBS and 1% penicillin–streptomycin. All cells in this work were cultured at 37 °C in conditions equilibrated with 5% CO_2_.

Human BMP‐2 and VEGF‐A plasmids were constructed by VectorBuilder Inc. Detailed construction maps of the plasmids used can be found in Figure S1A (Supporting Information). All plasmids were scaled up using plasmid kits (QIAGEN) following the manufacturer's protocols. The concentrations were obtained from NanoDrop 2000c Spectrophotometer (ThermoFisher Scientific).

### Cell Transfection Using Track‐Etched Membrane‐Based Nanoelectroporation (TM‐NanoEP) System

Cell transfection was carried out using a track‐etched membrane‐based nanoelectroporation (TM‐NanoEP) system. A track‐etched PET membrane was first treated with UV‐ozone to improve surface hydrophilicity, and the hAdMSCs were given 16–18 h to adhere to the membrane surface before transfection. The osteogenic‐angiogenic plasmid cocktail (BMP‐2 and VEGF‐A) was mixed at different weight ratios of BMP‐2 and VEGF‐A (BMP‐2 plasmids alone, 4:1, 2:1, and 1:1) with the concentration of BMP‐2 plasmids kept at 400 ng µL^−1^ in phosphate‐buffered saline (PBS) and loaded into a cured PDMS (Dow Corning) square (2.0 × 2.0 × 0.4 cm) with a hole of 12 mm in diameter in the middle. As illustrated in Figure 1A, an electric field with voltage ranging from 0 to 200 V (10 pulses at 10 ms per pulse with a 0.1 s interval) was applied between two Au‐plated electrodes to nanoporate the cell membrane and electrophoretically drive the plasmids into the cells. The transfected cells on the membrane were then incubated in α‐MEM (Gibco) with 2% exosome‐depleted fetal bovine serum (ThermoFisher Scientific) for 16–32 h at 37 °C.

### Phenotype Identification of hAdMSCs after TM‐nanoEP

The phenotype changes of hAdMSCs before and after TM‐NanoEP were identified by flow cytometry after staining with anti‐CD29, anti‐CD44, anti‐CD45, anti‐CD90, and anti‐CD105 antibodies, which were widely accepted as criteria for defining hAdMSCs. Briefly, the cell suspension of each group was divided into two aliquots (one for immunostaining and the other for unstained control) and was centrifuged at 2000 rpm for 5 min for cell collection. The cells were washed and resuspended with PBS, blocked with anti‐FcRII/III (human) antibody (BioLegend) for 30 min, and stained with anti‐CD29, anti‐CD44, anti‐CD45, anti‐CD90, and anti‐CD105 antibodies (BioLegend) on ice for 30 min. All data were analyzed using the Kaluza analysis software (Beckman Coulter).

### EV Purification and sEVs Isolation

Conditioned media were harvested and centrifuged at 2000×g for 30 min to remove cells and debris for downstream sEV isolation and purification (Figure 1A). A tangential flow filtration (TFF) system was used to enrich and diafiltrate sEVs from the collected media as previously reported.^[^ ^33^ ^]^ Briefly, a peristaltic pump (Cole–Parmer) circulates the media through a hollow fiber filter cartridge (Repligen). Diafiltration was then performed to remove the contaminants in the media through the circulation of PBS under a controlled flow rate (35 mL min^−1^). After TFF purification, the media was collected and further condensed to ≈5 mL by using an Amicon ultra‐centrifugal unit (Merck Millipore) with at 3000×g for 20 min. Then, the concentrated media was loaded into size exclusion columns (qEV10/70 nm, Izon Science), followed by elution with PBS buffer. The final sEV fractions were collected following the manufacturer's protocols and further condensed by using an Amicon ultra‐centrifugal unit at 3000×g for packaging and storage. All steps were performed at 4 °C.

### Characterization of Extracellular Vesicles (EVs) and Purified sEVs

The distribution and concentration of EVs and purified sEVs were analyzed using a tunable resistive pulse sensing (TRPS) technology (qNano Gold instrument, Izon Science) with NP150 (target size range of 40–150 nm) and NP300 (target size range of 150–600 nm) nanopore membranes for sEV fractions and large‐size particle fractions, respectively. The RNA yield and size distribution in the sEV fractions were analyzed using an Agilent 2100 Bioanalyzer with an RNA 6000 Pico kit (Agilent Technologies). Synthesized BMP‐2 and VEGF‐A mRNAs (100 ng each mRNA) were used for comparison.

### Microscopy Analysis of Cells and sEVs

Cell‐electron microscopy (Cell‐EM) analysis was collected 12 h after TM‐NanoEP, detached from the membrane by using a cell scraper, washed in PBS, and then fixed in 4% paraformaldehyde solution for 48 h. Samples were treated with 1% osmiumtetroxide (OsO_4)_ for 1 h at 4 °C before being dehydrated in increasing grades of alcohol (10 min each: 50%, 70%, 90%; 2 × 5 min: 100%) and embedded in epoxy resin. Ultrathin sections (70–100 nm) were cut with a Leica Ultracut UCT equipped with a diamond knife (Diatome), transferred to copper grids, stained with 2% aqueous uranyl acetate, and examined with a H‐7650 electron microscope at 80 kV.

For morphology analysis, 200 µL of the purified sEVs was first left on formvar/carbon copper grids, and droplets of the sEVs were cleared with filter papers. The grids were negatively stained with 2% uranyl acetate for 3 min. After washing with PBS, images were obtained by transmission electron microscopy (TEM) at 80 kV (H‐7650, Hitachi). In scanning electron microscopy (SEM), the purified sEVs were fixed with a 2% glutaraldehyde and 0.1 m sodium cacodylate solution for 3 h, followed by 1% osmium tetraoxide and 0.1 m sodium cacodylate for 2 h to increase contrast. The sEVs were then dehydrated with increasing grades of alcohol and dried with a CO_2_ critical point dryer. Finally, a 2 nm layer of gold was sputtered onto the sample, and the samples were imaged using an Apreo 2 SEM (ThermoFisher Scientific).

The structure of the sEVs was further analyzed by cryogenic electron microscopy (Cryo‐EM). Briefly, 10 µL of EV aliquot sample was applied to a glow‐discharged 300‐mesh R2.0/2.0 Quantifoil grid. The specimen grid was blotted by Whatman #1 filtration paper and then immediately plunged into liquid ethane to rapidly form a thin layer of amorphous ice by using a Vitrobot Mark IV system (ThermoFisher Scientific). The grid was transferred under liquid nitrogen to Glacios CryoTEM (ThermoFisher Scientific). Images were recorded by Felcon direct electron detector (ThermoFisher Scientific).

### Single sEV Biochip Assay and Total Internal Reflection Fluorescence (TIRF) Microscopy Imaging

A single sEV biochip was prepared for characterizing therapeutic mRNAs within the t‐sEVs.^[^ ^33b^ ^]^ Briefly, a glass coverslip (#D 263 m Glass, 24 × 75 mm rectangle, 0.15 mm thickness Schott AG, Mainz, Germany) was deposited with thin layers of 2 nm thick Ti and 10 nm thick Au using a Denton e‐beam evaporator (DV‐502A, Moorestown). Then, the Au‐coated glass was immersed into a linker solution containing β‐mercaptoethanol (βME, Sigma–Aldrich), PEG‐SH (#MPEG‐SH‐2000, Laysan Bio Arab), and biotin‐PEG‐SH (#PG2‐BNTH‐2k, Nanocs) (molar ratio = 95:3:2) in 200 proof ethanol (Fisher Scientific) for overnight. Next, 0.005% (w/v) streptavidin‐conjugated gold nanoparticles (NPs, Nanocs) in PBS were introduced into the wells for 2 h at room temperature (RT) on a rocker (24 rpm, Benchmark Scientific). After washing with PBS, the surface was incubated with a biotinylated capture antibody cocktail (20 µg mL^−1^ human CD63 Antibody (R&D Systems) and 20 µg mL^−1^ human CD9 Antibody (R&D Systems) (Table S8, Supporting Information) for 1 h at RT on the rocker.

Then, MB‐FISH (Molecular Beacon‐Fluorescence In Situ Hybridization) probes (listed 5′–3′) for the mRNA targets were encapsulated in cationic liposomal nanoparticles (CLN) as previous studies described. The MB‐FISH probes targeting human BMP‐2 (NM_0 01200.4) and human VEGF‐A (NM_0 010 25366.2) mRNAs used herein were FAM‐ACCGCG‐GCCACTTCCACCACG AATCCAT‐CGCGGT‐BHQ1 and CY3‐ACCGCG‐CAGGATGGCTTGAAGATGTAC‐CGCGGT‐BHQ2, respectively. The CLN was formulated with 1,2‐dioleoyl‐3‐trimethylammonium‐propane (DOTAP, Avanti Polar Lipids, AL, USA), Cholesterol (Sigma‐Aldrich), 1,2‐dioleoyl‐sn‐glycero‐3‐phosphocholine (DOPC, Avanti Polar Lipids), and Bis(1,2‐distearoyl‐sn‐glycero‐3‐phosphoethanolamine)‐N‐[(polyethylene glycol)−2000] (Bis‐DSPE PEG2000, Avanti Polar Lipids) at a 50:30:18:2 molar ratio. After loading and washing, 1 × 10^10^ mL^−1^ b‐sEVs, e‐sEV_PBS_, and t‐sEV_Bone RNAs_ were then captured on the biochip by fusion with the MB/lipid mixture for another 2 h incubation at 37 °C. After rinsing with PBS, the samples were imaged using the TIRF microscope (Nikon Eclipse Ti Inverted Microscope System).

### RT–qPCR of mRNA Expression Level in Transfected Cells and Therapeutic sEVs

The expression of BMP‐2 and VEGF‐A mRNAs in transfected hAdMSCs and sEVs were measured using RT–qPCR following manufacturers’ protocols. In brief, total cell RNAs were obtained using TRIzol reagent (Invitrogen), while total RNAs from sEVs were purified using total exosome isolation reagent (from cell culture media) (Invitrogen), total RNA purification kits (Norgen Biotek) and RNase‐free DNase I (ThermoFisher Scientific) to remove minor DNA plasmid residues. Then, a high‐capacity cDNA reverse transcription kit (Applied Biosystem) was used to reverse transcribed into complementary DNA (cDNA). At last, diluted cDNA was mixed with TaqMan universal PCR master mix (Takara, Tokyo, Japan), TaqMan probes (4331182, ThermoFisher Scientific), and RNase‐free water to perform RT‐qPCR. Synthesized BMP‐2 and VEGF‐A mRNAs were used to construct the standard curve. The concentrations used were 0.0001, 0.001, 0.01, 0.1, 1, and 10 ng µL^−1^. TaqMan probe assays used in this study were listed as follows: human BMP‐2 (Hs00154192_m1), human VEGF‐A (Hs00900055_m1), and GAPDH (Hs02786624_g1). All experiments were performed in triplicates.

### Intracellular Staining and Co‐Localization Analysis

Cells after TM‐nanoEP with plasmid cocktail were transferred to confocal dishes and fixed in a 4% formaldehyde solution. The fixed cells were permeabilized with 0.2% (v/v) Triton X‐100 (Sigma–Aldrich) for 5 min. For mRNA staining, cells were incubated with blocking buffer (R37520, ThermoFisher Scientific) for 1 h at RT and then incubated with 1 µm MB‐FISHs probes for BMP‐2 and VEGF‐A mRNAs for 1 h at 37 °C. Cells were then incubated with fluorescence‐labeled primary antibodies: anti‐Rab7 (Cell Signaling) in 1% BSA solution after blocking with 5% BSA in PBS solution for 1 h at RT. Fluorescent images of stained cells were taken by TIRF microscopy (N‐SIM S, Nikon) and quantitated for co‐localization by Image J (Fiji 2.9.0).

### Western Blot

Expression of markers on EVs/sEVs and target proteins in cells were analyzed by Western blot. Cells for investigations of the mTORC1‐autophagy axis were collected 12 h after the TM‐nanoEP process. Total proteins were extracted by lysed in RIPA buffer containing 1 mm phenylmethanesulfonyl fluoride (PMSF), incubated with sample buffer at 95 °C for 5 min, separated by SDS‐PAGE and then transferred onto a polyvinylidene difluoride (PVDF) membrane. After blocking with 5% non‐fat dry milk overnight, the membranes were incubated with primary antibodies at 4 °C for 16 h and then horseradish peroxidase (HRP)‐conjugated secondary goat anti‐rabbit or anti‐mouse antibody at 37 °C for 1 h (Table S8, Supporting Information). Immunodetection was performed and quantified by chemiluminescence (ECL) reagents (ThermoFisher Scientific) and Image Studio Lite V5.2 (Licor Biosciences).

### sEV/Exosome Uptake

sEVs were first labeled by PKH26 (Sigma–Aldrich). Afterward, target cells were incubated with PKH‐labeled sEVs at the concentration of 1 × 10^6^ particles/cell for 24 h. For observation, the cells were fixed in 4% paraformaldehyde for 10 min, followed by incubation with FITC‐phalloidin at 37 °C for 45 min and DAPI at room temperature for 10 min for cytoskeleton and nuclei staining, respectively. The internalization of sEVs was observed and quantified by confocal laser‐scanning microscopy (Olympus Corporation, Japan) and Image J (Fiji 2.9.0), respectively. mRNA translation in the recipient cells was then investigated by Western blot 72 h after sEV uptake.

### In Vitro Alkaline Phosphatase (ALP) Activity, Mineralization Staining, and Tube Formation Assay

Recipient cells were incubated with purified sEVs at the concentration of 1 × 10^6^ particles/cell. After 7‐day incubation, the hBMSCs were lysed with RIPA buffer, and alkaline phosphatase (ALP) activity was determined by ALP Assay Kit (ab83369, Abcam). According to the manufacturer's protocol, ALP staining was performed on day 7 using a BCIP/NBT alkaline phosphatase color development kit (Beyotime, China). At the end of the incubation time, cells were fixed with 4% paraformaldehyde and stained with 1% Alizarin Red (pH 4.2). Mineralization of hBMSCs was analyzed on day 14 using Alizarin red staining (ARS). An inverted microscope (TS100‐F, Nikon, Japan) was used to observe ALP and ARS staining.

Tube formation was assessed using an in vitro angiogenesis assay kit (EMD Millipore). HUVECs (1 × 10^5^ cells per well) were incubated in a 48‐well plate coated with Matrigel for 12 h in serum‐free DMEM and then incubated with sEVs for another 12 h. Image J (Fiji, 2.9.0) was used to measure the total tube length on the captured images (magnification, ×10) by an inverted microscope (TS100‐F, Nikon, Japan).

### Exosomal Small RNA Profiling and Osteogenic–Angiogenic Effects

Small RNA sequencing was conducted using adapters with unique molecular identifiers (UMI) to label each molecule prior to library construction. For the library construction, the quality and concentration of RNA were first assessed on Bioanalyzer 2100 with an RNA (Pico) chip 398 (Agilent Technologies) after total RNA was extracted from different sEV groups using the miRNeasy kit (QIAGEN). After that, electrophoretic separation on polyacrylamide gel electrophoresis (PAGE) gel was used for purification, and small RNA regions corresponding to the 18–30 nt bands in the ssRNA Ladder Marker (Takara) were separated. Then, a 5‐adenylated, 3‐blocked single‐stranded DNA adapter was linked to the 3′ end of the selected small RNA fragment. Next, the reverse transcription (RT) primers (Invitrogen) were then added into the solution and cross‐linked to the 3′ adapter of RNAs and excessive free 3′ adapter, followed by adding the 5′ adaptor to be linked to the 5′ end of the products. RT enzyme was added to reverse transcribe the small RNAs into complementary DNAs (cDNAs). The resulting cDNAs with both 3′ and 5′ adaptors were amplified with high‐fidelity polymerase. PCR products ranging from 110 to 130 bp were purified using PAGE gel to remove primer dimers and other byproducts. These products were subsequently denatured into single strands and then cyclized to yield single‐stranded circular DNA molecules. Through rolling cycle amplification, the single‐stranded circular DNA molecules were then replicated, and a DNA nanoball (DNB) containing multiple copies of DNA was generated. High‐quality DNBs are then loaded into patterned nanoarrays using high‐intensity DNA nanochip technique and sequenced through combinatorial Probe‐Anchor Synthesis (cPAS).

BGI data analysis platform (https://biosys.bgi.com/) was used to perform RNA profiling data analysis. Multiple hypothesis test corrections for *p*‐value of the difference test were made, and the false discovery rate (FDR) was controlled to determine the threshold of *p*‐value. Differential miRNA expression analysis was performed using the DEGseq. Those differential miRNAs met the criteria of the ANOVA significance analysis, with FDR ≤ 0.001 (or *q* value≤ 0.05) and the absolute value of fold change ≥ 1.5 considered as significantly differential miRNAs. Potential target genes of miRNAs of these differential miRNAs were predicted by databases (miRanda, TargetScan, and RNAhybrid) and classified functionally according to the function classification of gene ontology (GO) and Kyoto Encyclopedia of Genes and Genomes (KEGG) pathway annotation. These enrichment analyses employed the hypergeometric test to discern pathways/functions significantly overrepresented in candidate genes, using all genes set as background as background. The phyper function in R software was used for the enrichment, and the resulting P values were then corrected by the Bonferroni method, and a corrected *p*‐value ≤ 0.05 was taken as a threshold. GO terms or KEGG terms fulfilling this condition were defined as significantly enriched terms.

To investigate the osteogenic‐angiogenic effects of miRNAs, 20 nm mimics and inhibitors for 10 selected miRNAs were transiently transfected into hBMSCs and HUVECs using Lipofectamine (RNAiMAX, Invitrogen) according to the manufacturer's protocols. Sequence information of miRNA mimics and inhibitors is listed in Table S7 (Supporting Information). After 48‐h transfection, hBMSCs and HUVECs were cultured in osteogenic differentiation medium and endothelial cell growth medium for another 3 days, and RT‐qPCR was then performed to determine marker expression of osteogenesis (ALP, BMP‐2, and RUNX2) and angiogenesis (VEGF, PECAM, and eNOS). In vitro ALP activity and tube formation assay were investigated as mentioned before, while expression of BMP‐2, VEGF, and their downstream pathways was analyzed by Western blot.

### Preparation of Injectable PEGS‐A Hydrogels and PEGS‐A/sEVs Hydrogels

PEGS acrylate (PEGS‐A) hydrogels were prepared using a thiol‐Michael addition click reaction of a PEGS pre‐polymer. Before preparing the PEGS‐A hydrogel, the PEGS pre‐polymer with a PEG molar ratio at 30% was synthesized through a two‐step polycondensation according to our previous works.^[^ ^23^, ^34^ ^]^ Next, a PEGS‐A pre‐polymer was synthesized by activating the residual hydroxyl groups of the PEGS pre‐polymer using acryloyl chloride (TCI) and triethylamine (TEA, Aladdin, equimolar to acryloyl chloride). PEGS‐A hydrogels were then crosslinked by mixing the PEGS‐A pre‐polymer aqueous solution with dithiothreitol (DTT, Macklin) and borax (Aladdin) in the thiol‐Michael addition click reaction. As for the injectable PEGS‐A/sEVs hydrogels, 1 × 10^11^ purified sEVs (b‐sEVs, e‐sEV_PBS,_, and t‐sEVs_Bone RNAs_) were first mixed with the above PEGS‐A pre‐polymer solution at 4 °C and were then crosslinked with a specific ratio of DTT and borax. The comprehensive performance of the hydrogels for sEV immobilization was assessed by gelation time, injectability, mechanical stiffness, sEV distribution, and controllable release, which can be optimized by regulating the molar ratio of borax and DTT (1:1 1:2, 1:3, 1:4, and 1:5) and pre‐polymer concentration (10%, 20%, 30% 40%, and 50%).

### Characterization of Injectable PEGS‐A Hydrogels and PEGS‐A/sEVs Hydrogels

Fourier transform infrared (FT‐IR) spectroscopy (Nicolet 5700, ThermoFisher Scientific) was used to analyze the PEGS and PEGS‐A pre‐polymers, PEGS‐A, and PEGS‐A/sEVs. Nuclear magnetic resonance (NMR) spectroscopy (Bruker Avance II 600, Bruker Corporation, Switzerland) was used to analyze the PEGS and PEGS‐A pre‐polymers. The data obtained were further analyzed using MestReNova NMR software.

The rheological properties of the PEGS‐A/sEVs precursors were determined using a HAAKE rheometer (MARS 60, Germany) with a universal container for the colloid with a feeding ratio of DTT to Borax ranging from 2:1 to 4:1. The shear elastic (*G*’) and viscous (*G*’’) moduli were measured using time sweep (0–20 min), at the frequency of 1 Hz and 1% deformation at 20 °C. The gelation time was determined with a vial‐tilting method. The compression performance of the PEGS‐A/sEVs (Ø12 mm × 5 mm) was assessed by a universal mechanical material testing machine (AG‐2000A, Shimadzu, Japan). The in vitro release profile of sEVs from PEGS‐A hydrogel was evaluated and compared with traditional gelatin spongy (GS). At each time point, the supernatant was collected and replaced with fresh PBS. The amounts of released sEVs were quantified by qNano to plot the release curves.

Flow cytometry was used to detect macrophage phenotypes after incubation with Lipofectamine (MessengerMAX, Invitrogen) with synthetic mRNAs, growth factors, and different PEGS‐A/sEVs groups. After washing with PBS twice, cells were resuspended with cell staining buffer and blocked by rat anti‐mouse CD16/32 (Biolegend) at 4 °C for 5 min. Cells were then stained with Alexa Fluor 647 rat anti‐Mouse CD197 and PE rat anti‐Mouse CD206 to determine M1 and M2 phenotypes, respectively. The samples were analyzed on an Accuri C6 flow cytometer (BD) and data were analyzed using Kaluza analysis software (Beckman Coulter).

In vitro osteogenic‐angiogenic effects of PEGS‐A/sEVs hydrogels were evaluated by ALP activity (3 and 7 days), mineralization staining (14 days), and tube formation assay (12 h). RAW264.7, rBMSCs and HUVECs were co‐cultured with pure PEGS‐A, PEGS‐A/b‐sEVs, PEGS‐A/e‐sEVs_PBS_, and PEGS‐A/t‐sEVs_Bone RNAs_ for a specified time and the assays followed protocols mentioned in previous sections.

### Animal Surgery and Femur Defect Model

Six–eight weeks old male Sprague−Dawley (SD) rats (National Tissue Engineering Center, Shanghai, China) with an average weight of ≈300 g were used. The animal experiment in this study was approved by the Animal Research Committee of Sixth People's Hospital, Shanghai Jiao Tong University School of Medicine (License number: SCXK (Hu) 2018‐0004). All animal experiments and experimental procedures were approved by the Animal Ethics Committee (Ethics number: No. 20210418012). After anesthesia by intraperitoneal injection of sodium pentobarbital (3.5 mg per 100 g), a 3.0 mm diameter latero‐lateral channel with 5.0 mm in length was created perpendicular to the shaft axis to destroy cancellous trabecular bone by using a trephine bur at a slow speed irrigated in saline solution to avoid thermal necrosis. The rats were randomized into six groups, including pure PEGS‐A (*n* = 8), PEGS‐A/b‐sEVs (*n* = 8), PEGS‐A/e‐sEVs_PBS_ (*n* = 8), PEGS‐A/t‐sEVs_Bone RNAs_ (*n* = 11), gelatin sponge(GS)/t‐sEVs_Bone RNAs_ (*n* = 3). The polymer solution or GS scaffolds with 1 × 10^12^ sEVs were then injected or placed into the defects. After the implantation, PEGS‐A/t‐sEVs_Bone RNAs_ and GS/t‐sEVs_Bone RNAs_ with three rats in each cohort were imaged for in vivo fluorescence on day 14. At specific time intervals (1, 4, and 8 weeks), the remaining rats were sacrificed. For 1 week, two rats in each group were assigned to in vivo mRNA translation analyses, and the remaining femurs were assigned to micro‐computerized tomography (CT) analysis and histological studies (three rats for each interval).

### In Vivo Fluorescence Imaging

Rats with PKH26‐labeled sEVs within the hydrogels (PEGS‐A/t‐sEVs_Bone RNAs_ and GS/t‐sEVs_Bone RNAs_) were imaged with an in vivo imaging system (VISQUE InVivo Smart‐LF, Vieworks, Korea) on days 1, 3, 7, and 14. Hair on two lower legs was shaved prior to imaging. Quantitative analysis of red fluorescence intensity was performed by measuring the average radiation efficiency (photon sec^−1^ cm^−2^ sr^−1^) in a region of interest (ROI). Data were normalized to fluorescence intensity on day 1.

### Microfil Perfusion

After 4 and 8 weeks of implantation, the rats were perfused with MV‐122 Microfil (Flowtech) to observe blood vessel formation in the bone defect. To access the vasculature, the chest was opened, and the descending aorta was ligated, followed by an incision of the inferior vena cava. An angiocatheter was inserted into the left ventricle of the rat and perfused with heparinized saline before introducing 20 mL of microfil, delivered at a rate of 2 mL min^−1^. The samples were left to set overnight at 4 °C. The femoral head was removed and decalcified for analysis using microcomputed tomography (Micro‐CT).

### Microcomputed Tomography (Micro‐CT) Measurements

In the 4th and 8th week, the femur samples were removed and fixed in 4% paraformaldehyde for 3 days at 4 °C. Micro‐CT measurements (Skyscan 1172, Bruker, Germany) were applied for 3D rotation scanning of the femur samples. Samples were scanned at 9 µm pixel resolution and reconstructed into 3D models via VG Studio software (Volume Graphics, Germany) with GridRec algorithm. For analysis of the bone regeneration process within the defect, the central region of the 3.0 mm diameter defect was defined by drawing a circular contour as the area of measurement per slice to obtain a consistent volume of interest (VOI) and to avoid including the native bone margins. After 3D reconstruction, the bone volume fraction (BV/TV), trabecular thickness (Tb.Th.), vessel volume, and bone mineral density (BMD) were calculated by using auxiliary software (Scanco Medical AG, Switzerland).

### Histology and Immunohistochemistry Analysis

In the 1st week, tissues around the femur defect were extracted and fixed in 4% paraformaldehyde for 3 days at 4 °C. After being dehydrated through a graded ethanol series, cleared in xylene, and embedded in paraffin, the samples were sectioned into 4.5 mm slices with a slicer (CRYOSTAR NX50, ThermoFisher Scientific). All sections were incubated with 0.3% H_2_O_2_ and blocked in 5% bovine serum albumin. Next, sections were incubated with CY3‐conjugated BMP‐2 and FITC‐conjugated VEGF‐A antibodies at 4 °C overnight. The sections were counterstained with DAPI and images were captured.

For 4 and 8 weeks, samples after micro‐CT analysis were decalcified in 14% EDTA for 1 month before being dehydrated with a gradient of ethanol and embedded in paraffin, and the 5 µm thickness sections were sliced and stained for hematoxylin & eosin (H&E) and Masson's trichrome (MT) analysis. For immunostaining, tissue sections were subjected to antigen retrieval. Then the slides were incubated with 0.3% H_2_O_2_ and blocked in 5% bovine serum albumin. Anti‐CD31 (PECAM‐1) and anti‐osteocalcin (OCN) antibodies were used for staining. All staining slides were captured using a light microscope (Nikon E100, Japan). Microvessel density (MVD) was counted based on the brown‐staining single endothelial cells or cell clusters, while integrated optical density (IOD) to the corresponding cavity area was calculated using Image J (Fiji 2.9.0). Three representative areas of five slides were calculated.

### Statistical Analysis

Data are presented as mean ± standard deviation (SD) of triplicates unless otherwise indicated. Statistical analyses were performed GraphPad Prism (version 9.3) and Origin (version 8.0) software using a two‐tailed Student's *t*‐test for two‐sample comparison or one‐way ANOVA with Tukey's multiple comparisons post hoc test. In these analyses, significant differences were accepted at ^*^ *p* < 0.05, ^**^ *p* < 0.01, ^***^ *p* < 0.005, and ^****^ *p* < 0.001.

## Conflict of Interest

L.J.L. and A.S.L. are shareholders at Spot Biosystems Ltd. All other co‐authors declare no competing interests.

## Author Contributions

Y.M. and L.S. contributed equally to this work. L.J.L., Y.Y., Y.M., and L.S. designed the study and all experiments with feedback from A.S.L., D.J.H., C‐L.C., and E.R. Y.M., J.Z., and J.P. designed and produced TM‐nanoEP based sEVs. E.R., J.Z., and Y.M. isolated and purified the sEVs. Y.M., L.S., and Y.Y. led the animal studies with the help of R.Z. and A.Z. Y.M., C‐L.C., J.P., J.Z., and X.Y.R. conducted sEVs characterization with the help of X.W., H. L., K.W., H.X., and E.R. Y.M., L.S., X.Z., Y.L., C.‐L.C., and Z.H. conducted in vitro experiments. Y.M. and L.S. conducted exosomal RNA sequencing and analysis. L.J.L., Y.M., L.S., and Y.Y. wrote the paper with feedback from C. L., A.S.L., D.J.H., and E.R.

## Supporting information

Supporting Information

Supplemental Video 1

## Data Availability

The data that support the findings of this study are available from the corresponding author upon reasonable request.
