# Supplementary material for: Exosomal mRNAs for Angiogenic–Osteogenic Coupled Bone Repair
Source: Adv Sci (Weinh). 2023 Oct 17;10(33):2302622. doi: 10.1002/advs.202302622 (PMC10667797; doi:10.1002/advs.202302622)
Supplement: Supplementary file 1 — Supporting Information [file ADVS-10-2302622-s001.pdf]

ADVANCED SCIENCE
Open Access

Supporting Information

for *Adv. Sci.*, DOI 10.1002/advs.202302622

Exosomal mRNAs for Angiogenic–Osteogenic Coupled Bone Repair

Yifan Ma, Lili Sun, Jingjing Zhang, Chi-ling Chiang, Junjie Pan, Xinyu Wang, Kwang Joo Kwak, Hong Li, Renliang Zhao, Xilal Y. Rima, Chi Zhang, Anan Zhang, Yutong Liu, Zirui He, Derek Hansford, Eduardo Reategui, Changsheng Liu, Andrew S. Lee, Yuan Yuan and Ly James Lee**

Supplementary Materials for

Exosomal mRNAs for Angiogenic–Osteogenic Coupled Bone Repair

Yifan Ma[1,3#], Lili Sun[2#], Jingjing Zhang[3], Chi-ling Chiang[3], Junjie Pan[3], Xinyu Wang[3], Kwang Joo Kwak[3], Hong Li[3], Renliang Zhao[6], Xilal Y. Rima[3], Chi Zhang[7], Anan Zhang[2], Yutong Liu[2], Zirui He[2], Derek Hansford[1], Eduardo Reategui[3], Changsheng Liu[2], Andrew S. Lee[4,5], Yuan Yuan[2,3*] and L. James Lee[1,3 *]

[1]Department of Biomedical Engineering, The Ohio State University; Columbus OH 43210, USA

[2]Key Laboratory for Ultrafine Materials of Ministry of Education and Frontiers Science Center for Materiobiology and Dynamic Chemistry, East China University of Science and Technology, Shanghai 200237, PR China

[3]William G. Lowrie Department of Chemical and Biomolecular Engineering, The Ohio State University, Columbus OH 43210, USA

[4]School of Chemical Biology and Biotechnology, Peking University Shenzhen Graduate School, Shenzhen 518055, China.

[5]Institute for Cancer Research, Shenzhen Bay Laboratory, Shenzhen 518055, China.

[6]Department of Orthopedic Surgery and Shanghai Institute of Microsurgery on Extremities, Shanghai Jiao Tong University Affiliated Sixth People's Hospital, Shanghai, 200233 China

[7]College of Pharmacy, The Ohio State University, Columbus OH 43210, USA.

[*]Corresponding authors: Yuan Yuan (yyuan@ecust.edu.cn) and L. James Lee (lee.31@osu.edu)

[#] Yifan Ma and Lili Sun contributed equally to this work.

This PDF file includes:

Supplemental Fig. S1-7

Fig. S1. pDNA maps used in this study and hAdMSCs after TM-nanoEP process at different voltages.
Fig. S2. RNA standard curves or ladders, zeta potential and total RNA amount in different sEV cohorts, and TIRFM images of blank control.
Fig. S3. TSC1/2 knockdown and quantitative results of intracellular activities from Western blot analysis.
Fig. S4. Correlation between the pDNA concentration ratios of BMP-2 to VEGF-A used in TM-nanoEP and the actual mRNA ratios found in t-sEVs$_{\text{Bone RNAs}}$.
Fig. S5. Supplemental information of exosomal miRNA profiling.
Fig. S6. Characterization of customized PEGS-A/sEVs hydrogel.
Fig. S7. *In vivo* distribution of therapeutic sEVs (t-sEVs) and morphometric evaluation of bone regeneration.
Fig. S8. Expression of heat shock proteins (HSPs) and stress granules (SGs) within sEVs, and their biocompatibility.

Table. S1-S8

Table. S1. TSC1/2 siRNA sequences.
Table. S2. Fold change of miRNAs in sEVs from untreated hAdMSCs and TM-nanoEP stimulated hAdMSCs with PBS.
Table. S3. Fold change of miRNAs in sEVs from TM-nanoEP stimulated hAdMSCs with PBS and pDNA cocktail (BMP-2 and VEGF-A).
Table. S4. KEGG pathway enrichment of miRNA target genes (t-sEVs$_{\text{Bone RNAs}}$ vs. e-sEVs$_{\text{PBS}}$).
Table. S5. KEGG pathway enrichment of miRNA target genes (b-sEVs vs. e-sEVs$_{\text{PBS}}$).
Table. S6. Selected miRNAs related to TGF-β and VEGF signaling pathways.
Table. S7. Information of miRNA mimics and inhibitors.
Table. S8. Information of antibodies used in this work.

Video. S1

Video. S1. High mechanical performance of PGES-A/t-sEVs

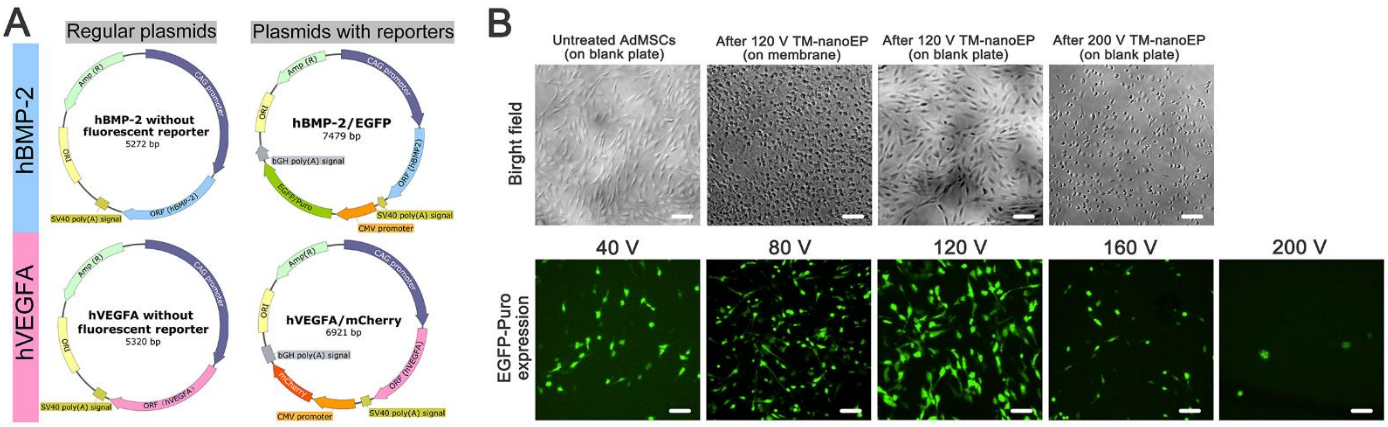

Supplemental Fig. 1| pDNA maps used in this study and hAdMSCs after TM-nanoEP process at different voltages. A) Plasmid maps of human BMP-2 and human VEGF-A with and without fluorescent reporters. **B)** Cell morphology and EGFP expression after TM-nanoEP under different voltages. Transfected hAdMSCs at 120V exhibited similar cell spreading to the untreated cells in the blank well. Cells transfected at 200V became unhealthy, and many detached after 24 h incubation. The transfection efficiency of EGFP at 6 h peaked at 120V (as depicted in Fig. 1B) and fell significantly at high voltages (160V and 200V). These results indicate cell responses, including cell transfection and EV secretion, are closely associated with cellular health (Scale bar: 100 µm).

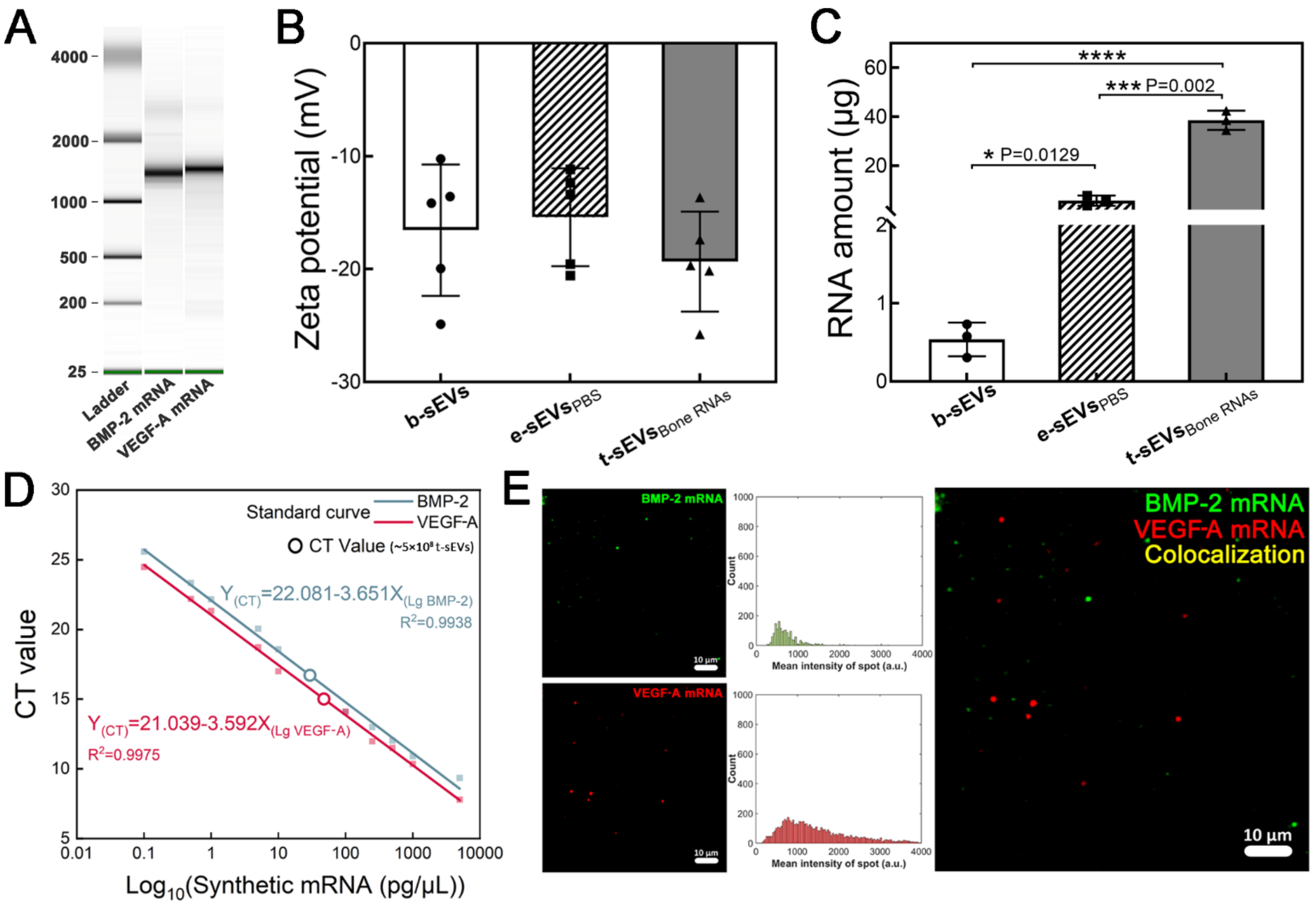

Supplemental Fig. 2| RNA standard curves or ladders, total RNA amount in different sEV cohorts and TIRFM images of blank control. A) Distribution of synthetic BMP-2 and VEGF-A mRNAs (100 ng each mRNA). The sizes of the two mRNAs are similar and around 1,500 nt. **B)** Zeta potential of b-sEVs, e-sEVs$_{PBS,}$ and t-sEVs$_{Bone\ RNAs}$. All the sEVs exhibit a negative charge, and there is no significant difference in zeta potential between transfected sEVs and native derived sEVs. **C)** RNA amount of b-sEVs, e-sEVs$_{PBS,}$ and t-sEVs$_{Bone\ RNAs}$ with the same sEV number (1×10^{12}). **D)** qPCR standard curves using synthetic mRNAs as internal standard. There are ~1.3 copies and ~1.8 copies of BMP-2 and VEGF-A per therapeutic sEV, respectively. **E)** Representative TIRFM images by using a single-sEV biochip for exosomal mRNA detection of b-sEVs from untreated hAdMSCs. Red dots: sEVs with VEGF-A mRNA; green dots: sEVs with BMP-2 mRNA; yellow dots: sEVs with both mRNAs (Scale bar: 10 μm). Few spots with fluorescence and weak mRNA expression are observed in b-sEVs, indicating blank sEVs carry few mRNAs. *$P < 0.05$, ***$P < 0.005$, ****$P < 0.0001$. All data are presented as mean ± SD. Student's t-test was used for comparison.

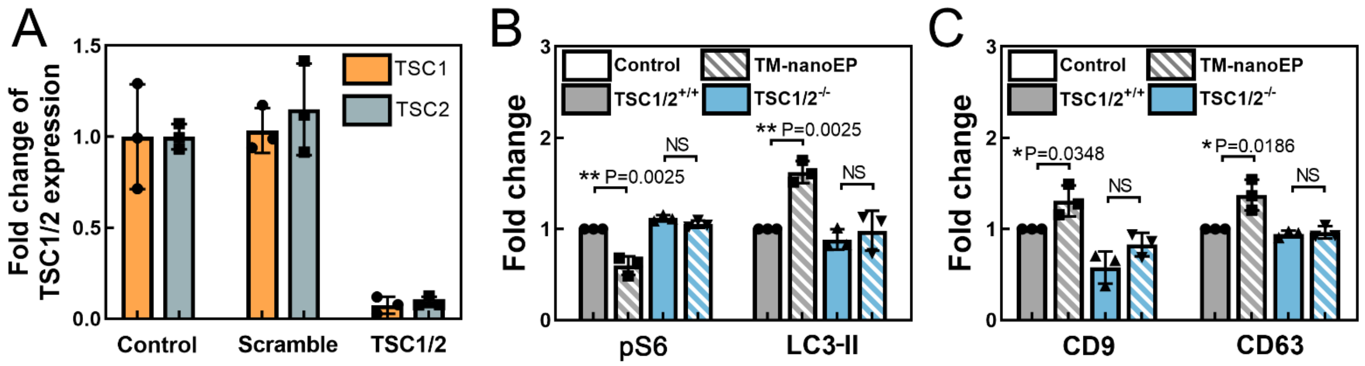

Supplemental Fig. 3| TSC1/2 knockdown and quantitative results of intracellular activities from Western blot analysis. A) Effects of tuberous sclerosis complex 1/2 (TSC1/2) knockdown on hAdMSCs. Quantitative Western blot analysis of **B)** mTORC1 and autophagy markers (pS6 and LC3-II), and **C)** intracellular sEV markers (CD9 and CD63). The results show TM-nanoEP has no discernible effect on mTORC1 and autophagic activities in TSC1/2$^{-/-}$ hAdMSCs, and there is no significant variation in exosomal markers within the cells following the TM-nanoEP process. *P < 0.05, **P< 0.01. All data are presented as mean ± SD. Student's t-test was used for comparison.

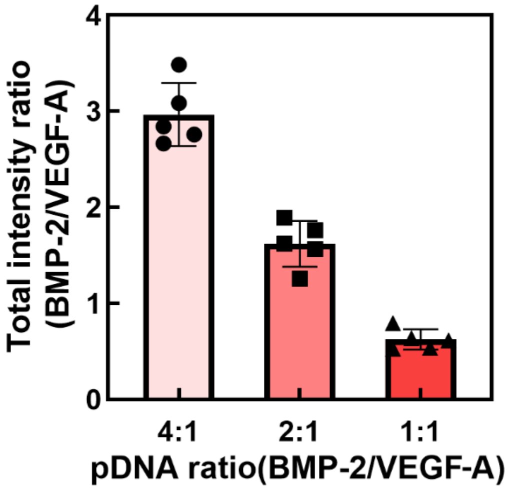

Supplemental Fig. 4| Correlation between the pDNA concentration ratios of BMP-2 to VEGF-A used in TM-nanoEP and the actual mRNA ratios found in t-sEVs$_{\text{Bone RNAs}}$ (n = 5). When the pDNA ratios used are 4:1, 2:1 and 1:1, the actual mRNA ratios of BMP-2 to VEGF-A are approximately 2.96:1, 1.60:1, and 0.62:1, respectively. The intrinsic VEGF mRNAs in sEVs from hAdMSCs could lead to decreased mRNA ratios in the sEVs.

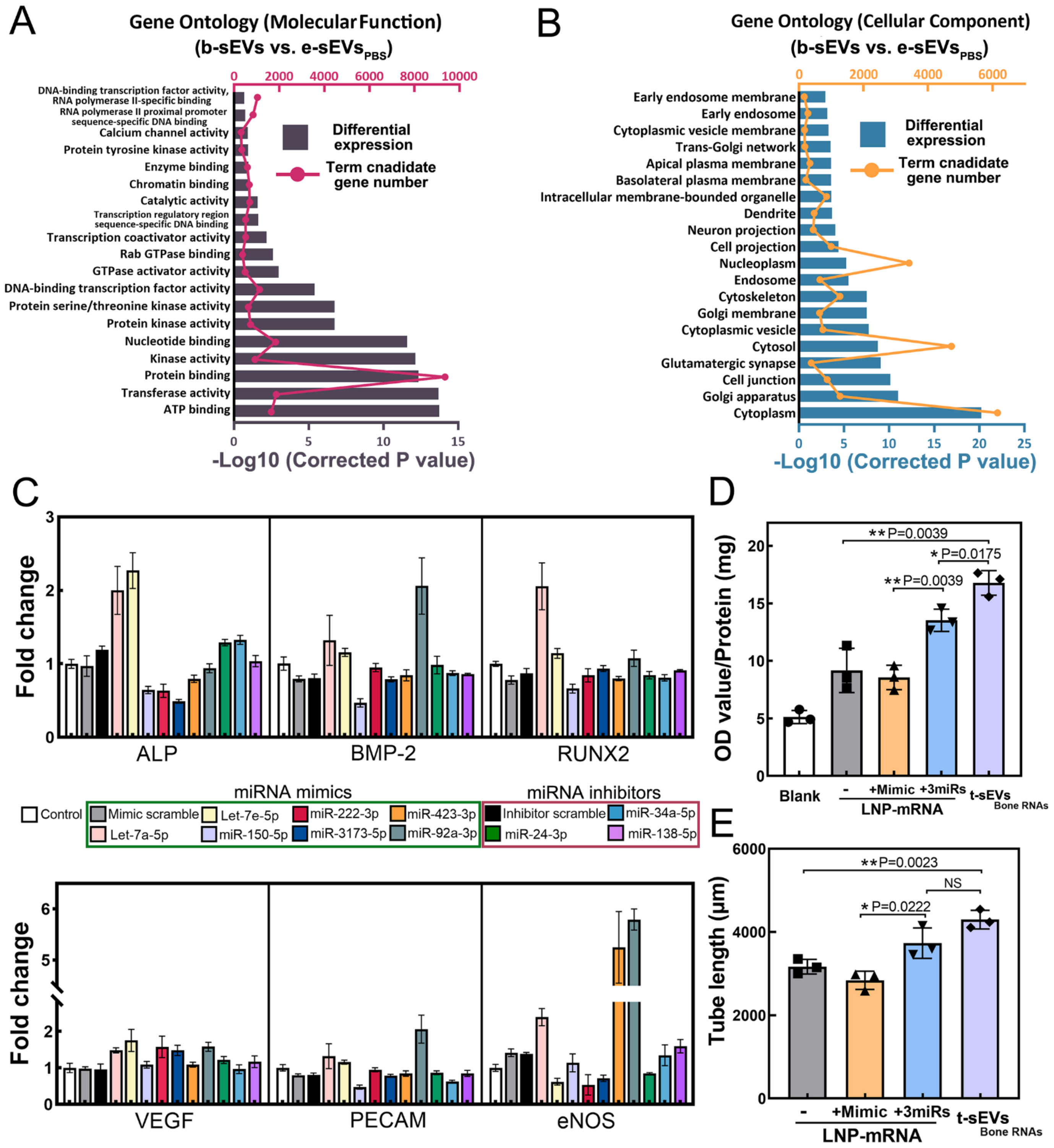

Supplemental Fig. 5| Supplemental information of exosomal microRNA (miRNA) profiling. Gene Ontology (GO) analyses of **A)** molecular function and **B)** cellular component (b-sEVs vs. e-sEVs$_{\text{PBS}}$). Q value < 0.05. **C)** 10 of the 29 miRNAs that are related to TGF-β and VEGF signaling pathways were selected for validation of osteogenic-angiogenic effects. Through RT-qPCR analysis, 3 of those 10 miRNAs, including let-7a-5p, let-7e-5p, and miR-92a-3p, could provide significantly improved osteogenic and angiogenic activities. Quantitative analyses of **H)** ALP activity of hBMSCs and **I)** tube formation of HUVECs cultured with LNP-mRNA, LNP-mRNA+Scr, LNP-mRNA+3miRs, and t-sEVs$_{\text{Bone RNAs}}$ for 7 days and 12 h (n = 3). $^*P < 0.05$, $^{**}P < 0.01$. All data are presented as mean ± SD. Student's t-test was used for comparison.

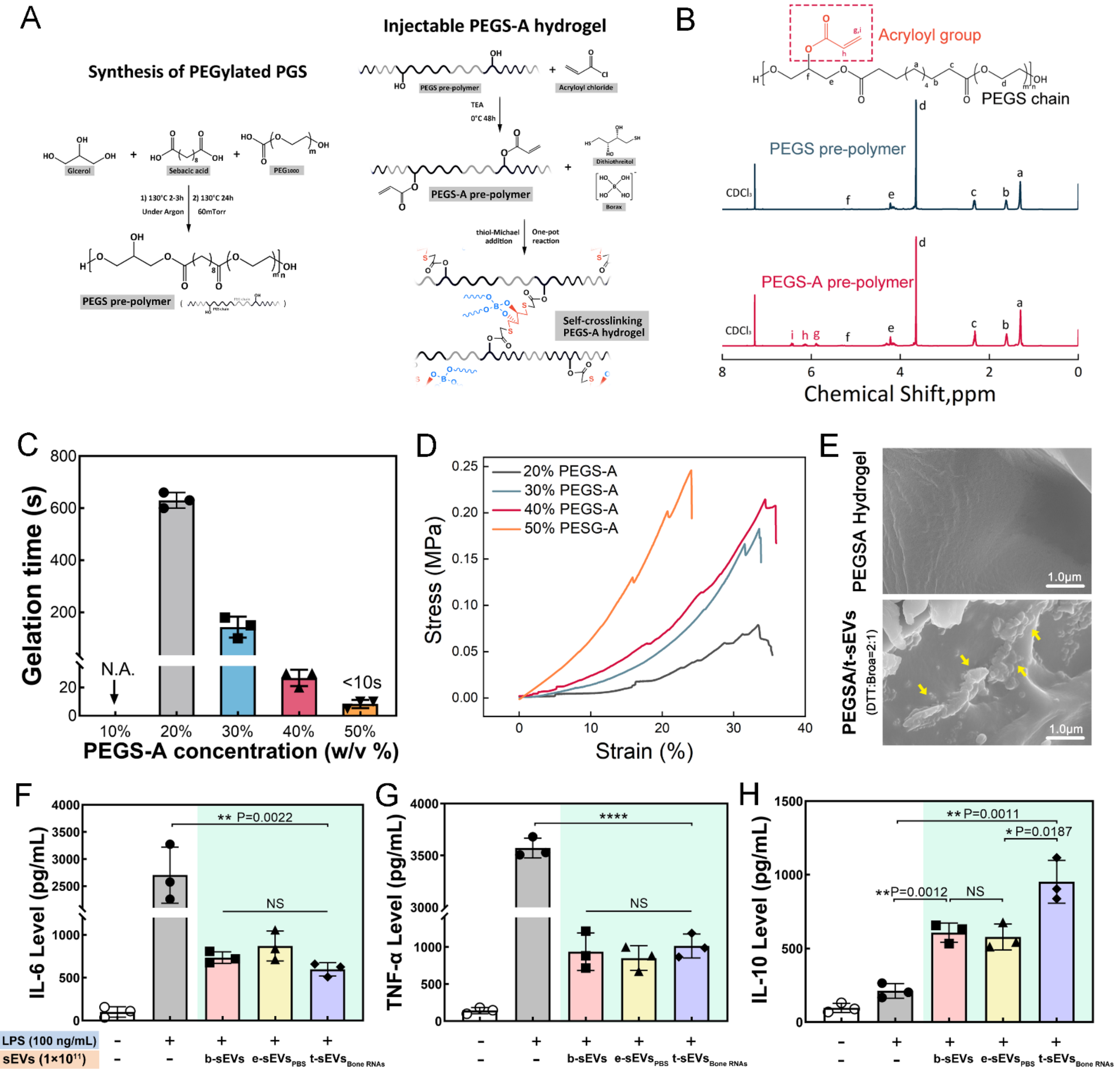

Supplemental Fig. 6| Characterization of PEGS-A/sEVs hydrogel. A) Synthesis schematics of PEGylated PGS (PEGS) and PEGS-A hydrogel. **B)** ^{1}H NMR spectra of PEGS and PESG-A pre-polymers. **C)** Gelation time of PEGS-A hydrogels with different PEGS-A pre-polymer concentrations when the ratio of DTT to Borax is 3:1. Among them, 10% PEGS-A pre-polymer is unable to crosslink, while gelation of 50% PEGS-A pre-polymer occurs in 10 s. **D)** Stress-strain curves of PEGS-A hydrogels with different PEGS-A pre-polymer concentrations when the ratio of DTT to Borax is 3:1. **E)** SEM images of PEGS-A hydrogel and PEGS-A/t-sEVs with a ratio of DTT to Borax at 2:1. Yellow arrows indicate aggregation of sEVs. **F)** IL-6, **G)** TNF-α, (M1 markers), and **H)** IL-10 (M2 marker) secretion in conditioned media of RAW264.7 macrophages (Mφs) assessed by ELISA after incubation with 100 ng/mL lipopolysaccharides (LPS) and sEVs (b-sEVs, e-sEVs$_{PBS}$, and t-sEVs$_{Bone\ RNAs}$). *P < 0.05, **P < 0.01 and ****P < 0.0001. All data are presented as mean ± SD. Student's t-test was used for comparison.

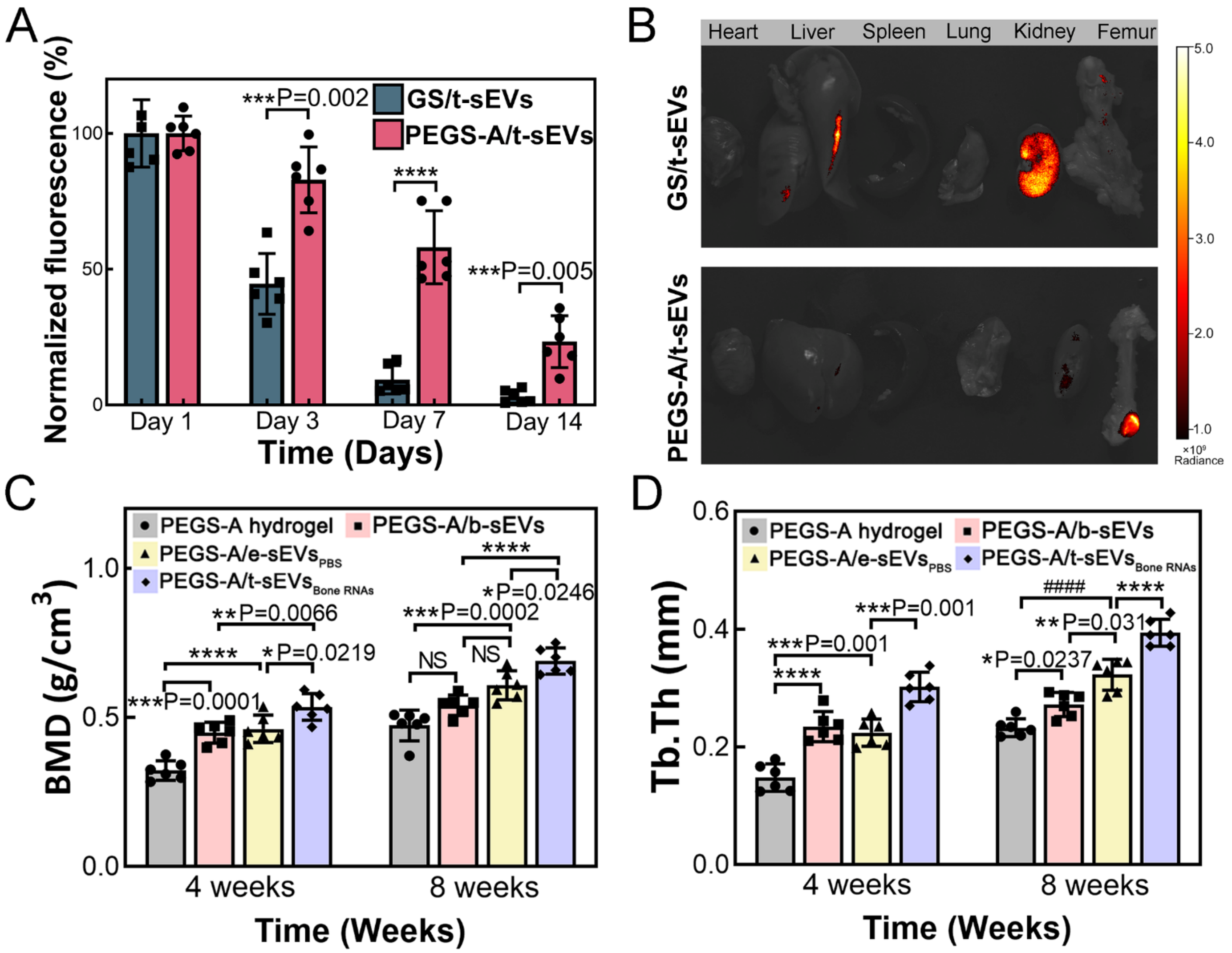

Supplemental Fig. 7| *In vivo* distribution of therapeutic sEVs (t-sEVs) and morphometric evaluation of bone regeneration. A) Quantification of fluorescence intensity over a 14-day time period. The results are normalized to the fluorescence intensity at day 1. **B)** SEV distribution in main organs and femur defects. The analyses show that sEVs encapsulated in PEGS-A hydrogels can be locally delivered with low hepatic, splenic, and renal accumulation. Morphometric analyses of **C)** bone mineral density (BMD) and **D)** trabecular thickness (Tb.Th.) for pure and PEGS-A/sEVs hydrogel cohorts at 4- and 8-week after injection. *P < 0.05, **P < 0.01, ***P < 0.005, ****P < 0.0001, ####P < 0.0001. All data are presented as mean ± SD. Student's t-test or one-way ANOVA was used for comparison.

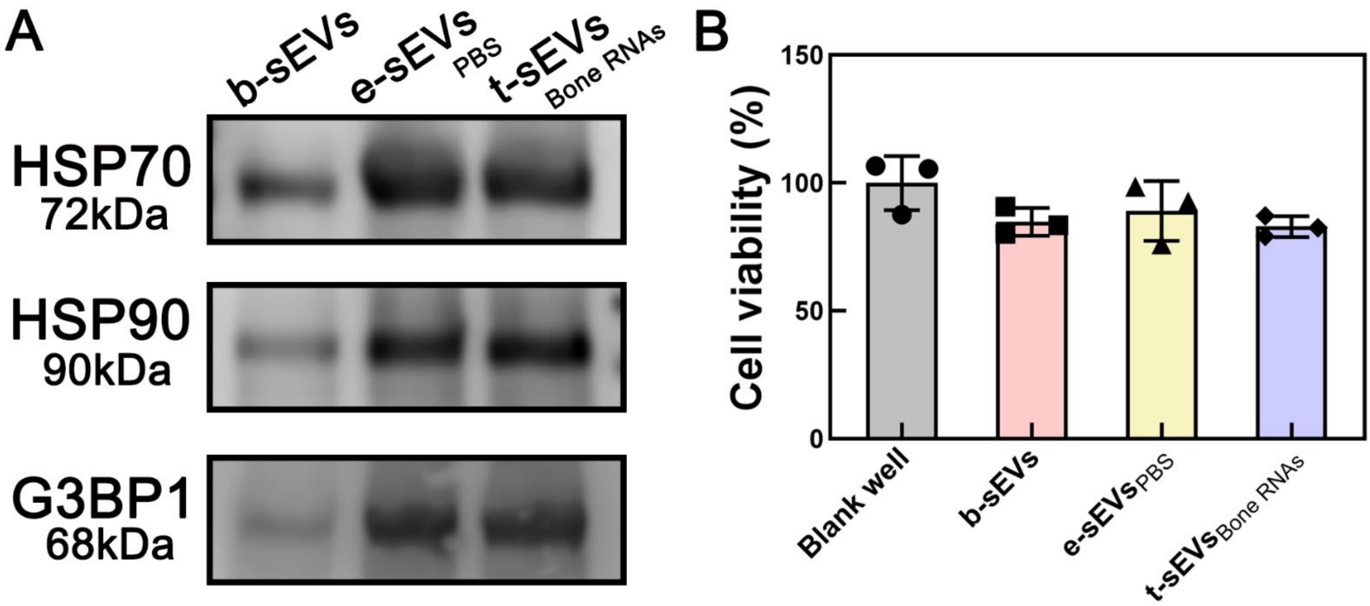

Supplemental Fig. 8| Expression of heat shock proteins (HSPs) and stress granules (SGs) within sEVs, and their biocompatibility. A) Western blot analyses of HSPs (HSP70 and HSP90) and SGs expression (using G3BP Stress Granule Assembly Factor 1 (G3BP1) as a marker) in different sEVs. Both e-sEVs$_{PBS}$ and t-sEVs$_{Bone\ RNAs}$ showed increased HSP expression when compared to the b-sEVs, suggesting the role of the thermal shock mechanism in TM-nanoEP-induced sEV biogenesis. Furthermore, these TM-nanoEP-induced sEVs demonstrated a significantly increased G3BP1 expression, indicating the presence of SG components in sEVs produced by TM-nanoEP. **B)** Cell viability of hBMSCs exposed to different sEVs at a dosage of 1×10^{6}/cell over a 3-day period. The sEVs produced by TM-nanoEP (e-sEVs$_{PBS}$ and t-sEVs$_{Bone}$ RNAs) showed no significant difference from the native sEVs (b-sEVs).

Table S1. | TSC1/2 siRNA sequences.

Target Gene	siRNA	Sequences
TSC-1	siTSC-1 sense	GCUGUCUUUAAAGAGAACCTT
	siTSC-1 anti-sense	GGUUCUCUUUAAAGACAGCTG
TSC-2	siTSC-2 sense	GCAGAGGGUAAACAGACGGAGUUUA
	siTSC-2 anti-sense	UAAACUCCGUCUGUUUACCCUCUGC
Scramble	Scramble sense	CCUAAGGUUAAGUCGCCCUCG
	Scramble anti-sense	CGAGGGCGACUUAACCUUAGG

Table S2. | Fold change of miRNAs in sEVs from untreated hAdMSCs and TM-nanoEP stimulated hAdMSCs with PBS.

	miRNA ID	Average read counts in b-sEVs	Average expression in b-sEVs	Average read counts in e-sEVs$_{PBS}$	Average expression in e-sEVs$_{PBS}$	Exosomal miRNA Log2 (Fold change e-sEVs$_{PBS}$ /b-sEVs)
Up-regulation	hsa-miR-423-5p	0	0.00	18.33	2.13	11.05
	hsa-miR-376a-3p	266.67	28.53	1520.67	180.15	2.66
	hsa-miR-329-3p	7.67	0.82	40.67	4.82	2.55
	hsa-miR-369-3p	14	1.48	72	8.52	2.52
	hsa-miR-381-3p	49	5.28	176	20.75	1.97
	hsa-miR-656-3p	16.67	1.78	53.67	6.36	1.84
	hsa-miR-199b-5p	112.33	12.05	330.33	39.12	1.70
	hsa-miR-138-5p	2251.67	12.78	11812.67	37.44	1.55
	hsa-miR-376b-3p	23.67	2.54	61.67	7.28	1.52
	hsa-miR-410-3p	46.67	5.01	112.33	13.29	1.41
	hsa-miR-376c-3p	321	34.45	761.67	90.10	1.39
	hsa-miR-154-5p	543	58.07	1135.33	134.21	1.21
	hsa-miR-146a-5p	772	83.55	1625.33	190.91	1.19
	hsa-miR-92a-3p	1597.33	172.88	3307	389.12	1.17
	hsa-miR-889-3p	53	5.71	101.67	12.01	1.07
	hsa-miR-378a-3p	1041.67	111.91	1826	215.52	0.95
	hsa-miR-221-3p	1026.33	110.10	1701.67	201.19	0.87
	hsa-miR-99a-5p	424.33	45.77	681.67	80.33	0.81
	hsa-miR-196a-5p	1153.33	123.70	1809	213.26	0.79
Down-regulation	hsa-miR-145-3p	18	1.92	0	0.00	-10.91
	hsa-miR-150-5p	925	97.40	9.33	1.10	-6.47
	hsa-miR-194-5p	27	2.85	0.33	0.04	-6.16
	hsa-miR-549a-5p	39	4.22	1.33	0.15	-4.78
	hsa-miR-125b-1-3p	26.67	2.88	1	0.12	-4.63
	hsa-miR-122-5p	872.67	93.69	32	3.78	-4.63

	hsa-miR-143-3p	7444	798.79	292	34.48	-4.53
	hsa-miR-145-5p	873.67	93.22	43	5.09	-4.20
	hsa-miR-139-5p	98	10.47	5.67	0.67	-3.97
	hsa-miR-374c-3p	57.67	6.19	4.67	0.56	-3.47
	hsa-miR-6529-5p	63.67	6.82	6.33	0.75	-3.18
	hsa-miR-3074-5p	3453	373.01	407.67	47.86	-2.96
	hsa-miR-1246	823.33	88.80	140.33	16.54	-2.42
	hsa-miR-189-5p	204.33	21.99	37	4.34	-2.34
	hsa-let-7i-5p	11812.67	1268.39	2251.67	265.48	-2.26
	hsa-miR-27b-3p	3629.67	390.03	726.67	85.69	-2.19
	hsa-miR-152-3p	96.33	10.32	19.33	2.29	-2.17
	hsa-miR-151a-5p	563.67	60.56	117.33	13.85	-2.13
	hsa-miR-451a	2540.33	271.30	548.67	64.45	-2.07
	hsa-miR-24-3p	16482	1781.04	3739.67	439.44	-2.02
	hsa-miR-126-3p	498.67	53.43	118	13.87	-1.95
	hsa-miR-181a-5p	2952.33	316.70	817.33	96.15	-1.72
	hsa-miR-127-3p	2781.33	299.39	938.33	110.57	-1.44
	hsa-miR-21-5p	2631.33	282.53	886.33	104.55	-1.43
	hsa-miR-26a-5p	4109.33	440.35	1398.33	164.77	-1.42
	hsa-miR-23a-3p	3419.33	366.86	1241	146.18	-1.33

Table S3. | Fold change of miRNAs in sEVs from TM-nanoEP stimulated hAdMSCs with PBS and pDNA cocktail (BMP-2 and VEGF-A).

	miRNA ID	Average read counts in e-sEVs$_{PBS}$	Expression of e-sEVs$_{PBS}$	Average read counts in t-sEVs$_{Bone\ RNAs}$	Expression of t-sEVs$_{Bone\ RNAs}$	Exosomal miRNA Log2 (Fold change of t-sEVs$_{Bone\ RNAs}$/ e-sEVs$_{PBS}$)
Up-regulation	hsa-miR-3173-5p	0	0.00	53.67	4.33	12.08
	hsa-miR-150-5p	9.33	1.10	2586.33	188.70	7.42
	hsa-miR-204-5p	0.67	0.08	73	5.81	6.22
	hsa-miR-1246	140.33	16.54	8657	718.27	5.44
	hsa-miR-10a-5p	98	11.55	3142	258.08	4.48
	hsa-miR-337-3p	132.33	15.63	2730.33	223.79	3.84
	hsa-miR-323a-3p	26	3.09	511.33	42.90	3.80
	hsa-miR-615-3p	10.67	1.26	192.33	15.87	3.65
	hsa-miR-328-3p	21.67	2.55	348	27.87	3.45
	hsa-miR-10b-5p	100.33	11.88	1417.33	117.73	3.31
	hsa-let-7e-5p	90	10.67	1266.33	104.11	3.29
	hsa-miR-423-3p	812.33	95.63	11024.33	898.74	3.23
	hsa-let-7d-3p	37.67	4.42	491.33	39.15	3.15
	hsa-miR-424-3p	15.67	1.86	166.33	13.61	2.87
	hsa-miR-92b-3p	143	16.85	1436.67	113.23	2.75
	hsa-miR-1180-3p	14.67	1.73	131.33	10.55	2.61
	hsa-miR-189-5p	37	4.34	281	22.86	2.40
	hsa-miR-99b-5p	220	25.86	1609.67	132.92	2.36
	hsa-let-7a-5p	3363	397.31	22978	1897.99	2.26
	hsa-miR-92a-3p	3307	389.12	22422.33	1857.95	2.26
	hsa-miR-31-5p	30.33	3.59	205	16.68	2.22
	hsa-miR-222-3p	2359.67	277.06	14063	1168.95	2.08
	hsa-miR-484	72	8.51	421	35.41	2.06

	has-miR-264-3p	1175.67	138.27	6631.33	557.97	2.01
	hsa-let-7d-5p	186.67	22.04	1075.67	88.09	2.00
	hsa-miR-127-3p	938.33	110.57	5383.33	436.73	1.98
	hsa-let-7f-5p	1268	149.57	6400	525.11	1.81
	hsa-miR-574-5p	185	21.76	911.67	74.67	1.78
Down-regulation	hsa-miR-671-5p	12.67	1.50	0	0.00	-10.55
	hsa-miR-1287-5p	9.33	1.10	0	0.00	-10.10
	hsa-miR-21-3p	8.67	1.02	0	0.00	-9.99
	hsa-miR-34c-3p	19.33	2.31	0.333	0.03	-6.26
	hsa-miR-4659a-3p	12.333	1.46	0.667	0.06	-4.73
	hsa-miR-193a-3p	65.667	7.76	4	0.33	-4.56
	hsa-miR-4488	20	2.37	2	0.15	-4.02
	hsa-miR-374b-5p	26	3.06	2.67	0.22	-3.81
	hsa-miR-655-3p	120	14.25	15.33	1.12	-3.67
	hsa-miR-193b-3p	1011.67	119.51	106.67	9.47	-3.66
	hsa-miR-154-5p	1135.33	134.21	293.33	25.44	-2.40
	hsa-miR-138-5p	318.33	37.44	105	8.89	-2.07
	hsa-miR-376c-3p	761.67	90.10	275.33	22.85	-1.98
	hsa-miR-382-5p	287.33	33.97	112.33	9.55	-1.83
	hsa-miR-148a-3p	54	6.34	23	1.79	-1.83
	hsa-miR-27a-3p	4246.67	500.74	2001.67	171.86	-1.54
	hsa-miR-487b-3p	152	17.99	78.33	6.52	-1.46
	hsa-miR-27b-3p	726.67	85.69	393	33.40	-1.36
	hsa-miR-24-3p	3739.67	439.44	2102.33	179.91	-1.29
	hsa-miR-493-3p	165.67	19.44	101.33	8.28	-1.23
	hsa-miR-532-5p	247.67	29.17	156.33	13.13	-1.15

	hsa-miR-34a-5p	10309.33	1212.67	7512	620.86	-0.97
	hsa-miR-378a-3p	1826	215.52	1353	111.99	-0.94
	hsa-miR-22-3p	2876	338.60	2436	198.51	-0.77

Table S4. | KEGG pathway enrichment of miRNA target genes (t-sEVs$_{Bone\ RNAs}$ vs. e-sEVs$_{PBS}$). Two signaling pathways (TGF-β signaling pathway and VEGF signaling pathway) are directly associated with the introduced bone plasmid cocktail.

KEGG Pathway Term Description	KEGG Pathway Term Level	Term Candidate Gene Number	Corrected P value (P<0.05)
MAPK signaling pathway	Signal transduction	292	1.04E-08
Rap1 signaling pathway	Signal transduction	207	9.34E-06
AMPK signaling pathway	Signal transduction	119	2.65E-04
Axon guidance	Development and regeneration	177	3.86E-04
ErbB signaling pathway	Signal transduction	85	5.03E-04
Calcium signaling pathway	Signal transduction	188	6.37E-04
MAPK signaling pathway - fly	Signal transduction	80	7.88E-04
Phospholipase D signaling pathway	Signal transduction	145	9.59E-04
Osteoclast differentiation	Development and regeneration	125	9.64E-04
Phosphatidylinositol signaling system	Signal transduction	98	1.47E-03
Ras signaling pathway	Signal transduction	224	1.58E-03
Sphingolipid signaling pathway	Signal transduction	117	1.76E-03
Hippo signaling pathway - fly	Signal transduction	69	2.11E-03
Hippo signaling pathway	Signal transduction	148	2.76E-03
HIF-1 signaling pathway	Signal transduction	107	3.68E-03
PI3K-Akt signaling pathway	Signal transduction	336	8.48E-03
cAMP signaling pathway	Signal transduction	207	9.21E-03
Foxo signaling pathway	Signal transduction	127	1.06E-02
Hedgehog signaling pathway	Signal transduction	50	1.16E-02
Wnt signaling pathway	Signal transduction	153	1.47E-02
mTOR signaling pathway	Signal transduction	146	2.16E-02
cGMP-PKG signaling pathway	Signal transduction	160	2.25E-02
NF-kappa B signaling pathway	Signal transduction	97	2.46E-02
TNF signaling pathway	Signal transduction	108	3.30E-02
VEGF signaling pathway	Signal transduction	58	3.38E-02
TGF-β signaling pathway	Signal transduction	91	3.58E-02

Table S5. | KEGG pathway enrichment of miRNA target genes (e-sEVs$_{PBS}$ vs. b-sEVs). Two signaling pathways (TGF-β signaling pathway and VEGF signaling pathway) directly associated with the introduced bone plasmid cocktails.

KEGG Pathway Term Description	KEGG Pathway Term Level	Term Candidate Gene Number	Corrected P value (P<0.05)
Axon guidance	Development and regeneration	175	1.22E-07
MAPK signaling pathway	Signal transduction	275	4.11E-06
Rap1 signaling pathway	Signal transduction	198	1.07E-05
Osteoclast differentiation	Development and regeneration	121	1.45E-04
Axon regeneration	Development and regeneration	89	1.87E-04
Hippo signaling pathway	Signal transduction	143	2.66E-04
Phospholipase D signaling pathway	Signal transduction	139	4.13E-04
Calcium signaling pathway	Signal transduction	179	4.70E-04
Hippo signaling pathway - fly	Signal transduction	67	8.88E-04
TNF signaling pathway	Signal transduction	106	8.81E-04
AMPK signaling pathway	Signal transduction	113	1.09E-03
HIF-1 signaling pathway	Signal transduction	103	1.25E-03
Sphingolipid signaling pathway	Signal transduction	112	1.22E-03
mTOR signaling pathway	Signal transduction	141	1.86E-03
Wnt signaling pathway	Signal transduction	147	2.23E-03
cAMP signaling pathway	Signal transduction	197	2.82E-03
Ras signaling pathway	Signal transduction	211	2.94E-03
Phosphatidylinositol signaling system	Signal transduction	93	3.87E-03
FoxO signaling pathway	Signal transduction	121	6.00E-03
cGMP-PKG signaling pathway	Signal transduction	152	1.03E-02
Hedgehog signaling pathway	Signal transduction	48	1.19E-02
ErbB signaling pathway	Signal transduction	79	1.71E-02
PI3K-Akt signaling pathway	Signal transduction	313	2.35E-02
MAPK signaling pathway - fly	Signal transduction	74	2.82E-02
VEGF signaling pathway	Signal transduction	55	4.00E-02
TGF-β signaling pathway*	Signal transduction*	85*	7.21E-02* (P>0.05)

* TGF-β signaling pathway does not show significant enrichment.

Table. S6. | Selected miRNAs related to TGF-β and VEGF signaling pathways. Based on the fold change, expression level, and predicted targeted gene nodes within the TGF-β (BMP-2)/VEGF pathways, 10 of the 29 miRNAs were selected and labeled in blue.

miRNA ID	Expression in sEVs		Fold change	Target gene nodes in TGF-beta and VEGF pathways
	e-sEVs$_{PBS}$	t-sEVs$_{BoneRNAs}$		
hsa-let-7a-5p	397.31	1897.99	2.26	4
hsa-let-7e-5p	10.67	104.11	3.29	4
hsa-miR-138-5p	37.44	8.89	-2.07	14
hsa-miR-150-5p	1.10	188.70	7.42	7
hsa-miR-222-3p	277.06	1168.95	2.08	4
hsa-miR-24-3p	439.44	179.91	-1.29	9
hsa-miR-3173-5p	0.00	4.33	12.08	12
hsa-miR-34a-5p	1212.67	620.86	-0.97	15
hsa-miR-423-3p	95.63	898.74	3.23	9
hsa-miR-92a-3p	389.12	1857.95	2.26	5
hsa-let-7d-5p	22.04	88.09	2.00	6
hsa-let-7f-5p	149.57	525.11	1.81	1
hsa-miR-1180-3p	1.73	10.55	2.61	13
hsa-miR-127-3p	110.57	436.73	1.98	1
hsa-miR-148a-3p	6.34	1.79	-1.83	3
hsa-miR-193a-3p	7.76	0.33	-4.56	1
hsa-miR-204-5p	0.08	5.81	6.22	4
hsa-miR-27a-3p	500.74	171.86	-1.54	7
hsa-miR-27b-3p	85.69	33.40	-1.36	7
hsa-miR-31-5p	3.59	16.68	2.22	3
hsa-miR-323a-3p	3.09	42.90	3.80	2
hsa-miR-328-3p	2.55	27.87	3.45	14
hsa-miR-378a-3p	215.52	111.99	-0.94	3
hsa-miR-382-5p	33.97	9.55	-1.83	3
hsa-miR-484	8.51	35.41	2.06	8
hsa-miR-493-3p	19.44	8.28	-1.23	2
hsa-miR-532-5p	29.17	13.13	-1.15	1
hsa-miR-574-5p	21.76	74.67	1.78	8
hsa-miR-615-3p	1.26	15.87	3.65	7

Table. S7. | Information of miRNA mimics and inhibitors.

	miRNA	Sequences
Mimics	has-let-7a-5p F	UGAGGUAGUAGGUUGUAUAGUU
	has-let-7a-5p R	AACUAUACAACCUACUACCUCA
	has-let-7e-5p F	UGAGGUAGGAGGUUGUAUAGUU
	has-let-7e-5p R	AACUAUACAACCUCCUACCUCA
	hsa-miR-150-5p F	UCUCCCAACCCUUGUACCAGUG
	hsa-miR-150-5p R	CACUGGUACAAGGGUUGGGAGA
	has-miR-222-3p F	AGCUACAUCUGGCUACUGGGU
	has-miR-222-3p R	ACCCAGUAGCCAGAUGUAGCU
	has-miR-3173-5p F	UGCCCUGCCUGUUUUCUCCUUU
	has-miR-3173-5p R	AAAGGAGAAAACAGGCAGGGCA
	hsa-miR-423-3p F	AGCUCGGUCUGAGGCCCCUCAGU
	hsa-miR-423-3p R	ACUGAGGGGCCUCAGACCGAGCU
	hsa-miR-92a-3p F	UAUUGCACUUGUCCCGGCCUGU
	hsa-miR-92a-3p R	ACAGGCCGGGACAAGUGCAAUA
	Mimic NC F	UCACAACCUCCUAGAAAGAGUAGA
	Mimic NC R	UCUACUCUUUCUAGGAGGUUGUGA
Inhibitors	hsa-miR-27a-3p	GCGGAACUUAGCCACUGUGAA
	hsa-miR-34a-5p	ACAACCAGCTAAGACACUTCCA
	hsa-miR-138-5p	CGGCCUGAUUCACAACACCAGCU
	Inhibitor NC	UCUACUCUUUCUAGGAGGUUGUGA

Table. S8. | Information of antibodies used in this work.

Target protein purpose	Host	Manufacture	Cat.	Working concentration
β-actin WB	Rabbit	Cell signaling	#4970	1:1000 in TBST
GAPDH WB	Rabbit	Abcam	ab9483	1:1000 in TBST
CD9 WB	Rabbit	Abcam	ab92726	1:1000 in TBST
CD63 WB	Rabbit	Abcam	ab231975	1:1000 in TBST
Arf6 WB	Rabbit	Cell signaling	#5740	1:1000 in TBST
S6 Ribosomal Protein WB	Rabbit	Cell signaling	#2217	1:1000 in TBST
Phospho-S6 Ribosomal Protein WB	Rabbit	Cell signaling	#35708	1:2000 in TBST
LC3-II WB	Rabbit	Cell signaling	#3868	1:1000 in TBST
BMP-2 WB	Rabbit	Abcam	ab14933	1:500 in TBST
VEGF-A WB	Rabbit	Cell signaling	#50661	1:2000 in TBST
Phospho-AKT WB	Rabbit	Cell signaling	#4060	1:2000 in TBST
AKT WB	Rabbit	Cell signaling	#4685	1:1000 in TBST
Smad1/5/9 WB	Rabbit	Abcam	ab300164	1:1000 in TBST
Phospho-Smad1/5/9 WB	Rabbit	Cell signaling	#13820	1:1000 in TBST
p44/42 MAPK (Erk1/2) WB	Rabbit	Cell signaling	#4695	1:1000 in TBST
Phospho-p44/42 MAPK (Erk1/2) WB	Rabbit	Cell signaling	#4370	1:2000 in TBST
Heat shock protein 70 (HSP70)	Mouse	Invitrogen	MA3-006	1:1000 in TBST
Heat shock protein 90 (HSP90)	Rabbit	Cell Signaling	#4877	1:1000 in TBST
G3BP1	Rabbit	Cell Signaling	#17798	1:1000 in TBST
Rab7 Alexa Fluor 647 Conjugate IF	Rabbit	Cell Signaling	94298S	1:100 in 1% BSA
CD63 Capture antibody	Mouse	R&D Systems	#MAB5048	20 μg/mL in PBS
CD9 Capture antibody	Mouse	R&D Systems	#MAB1880	20 μg/mL in PBS
CD206 (PE) FC	Rat	BD bioscience	568273	1:200 in cells-PBS
CD197 (APC) FC	Rat	BD bioscience	560766	1:200 in cells-PBS
BMP-2 IF	Rabbit	Servicebio	GB12252	1:1000 in PBS
VEGF-A IF	Mouse	Servicebio	GB13034	1:1000 in PBS
CD31 (PECAM-1) IHC	Rabbit	Servicebio	GB11063-2	1:400 in PBS
Osteocalcin (OCN) IHC	Rabbit	Servicebio	GB11233	1:100 in PBS

Video. S1. High mechanical performance of PGES-A/t-sEVs when the PEGS-A precursor is formulated with a 30% pre-polymer concentration and a DTT/Borax ratio of 3:1.
